# The archaeome in metaorganism research, with a focus on marine models and their bacteria–archaea interactions

**DOI:** 10.3389/fmicb.2024.1347422

**Published:** 2024-02-27

**Authors:** Avril J. E. von Hoyningen-Huene, Corinna Bang, Philipp Rausch, Malte Rühlemann, Hanna Fokt, Jinru He, Nadin Jensen, Mirjam Knop, Carola Petersen, Lara Schmittmann, Thorsten Zimmer, John F. Baines, Thomas C. G. Bosch, Ute Hentschel, Thorsten B. H. Reusch, Thomas Roeder, Andre Franke, Hinrich Schulenburg, Eva Stukenbrock, Ruth A. Schmitz

**Affiliations:** ^1^Institute for General Microbiology, Kiel University, Kiel, Germany; ^2^Institute of Clinical Molecular Biology, Kiel University, Kiel, Germany; ^3^Hannover Medical School, Institute for Medical Microbiology and Hospital Epidemiology, Hannover, Germany; ^4^Section of Evolutionary Medicine, Institute for Experimental Medicine, Kiel University, Kiel, Germany; ^5^Max Planck Institute for Evolutionary Biology, Plön, Germany; ^6^Cell and Developmental Biology, Zoological Institute, Kiel University, Kiel, Germany; ^7^Department of Molecular Physiology, Zoology, Kiel University, Kiel, Germany; ^8^Evolutionary Ecology and Genetics, Zoological Institute, Kiel University, Kiel, Germany; ^9^Research Unit Ocean Dynamics, GEOMAR Helmholtz Institute for Ocean Research Kiel, Kiel, Germany; ^10^Marine Evolutionary Ecology, GEOMAR Helmholtz Center for Ocean Research, Kiel, Germany; ^11^Research Unit Marine Symbioses, GEOMAR Helmholtz Centre for Ocean Research Kiel, Kiel, Germany; ^12^Christian-Albrechts-Universität Kiel, Kiel, Germany; ^13^German Center for Lung Research (DZL), Airway Research Center North (ARCN), Kiel, Germany; ^14^Antibiotic Resistance Group, Max-Planck Institute for Evolutionary Biology, Plön, Germany; ^15^Environmental Genomics, Christian-Albrechts University of Kiel, Kiel, Germany

**Keywords:** archaeome, microbiome, metaorganism, host-associated microbiota, marine archaea, microbial community

## Abstract

Metaorganism research contributes substantially to our understanding of the interaction between microbes and their hosts, as well as their co-evolution. Most research is currently focused on the bacterial community, while archaea often remain at the sidelines of metaorganism-related research. Here, we describe the archaeome of a total of eleven classical and emerging multicellular model organisms across the phylogenetic tree of life. To determine the microbial community composition of each host, we utilized a combination of archaea and bacteria-specific 16S rRNA gene amplicons. Members of the two prokaryotic domains were described regarding their community composition, diversity, and richness in each multicellular host. Moreover, association with specific hosts and possible interaction partners between the bacterial and archaeal communities were determined for the marine models. Our data show that the archaeome in marine hosts predominantly consists of *Nitrosopumilaceae* and *Nanoarchaeota*, which represent keystone taxa among the porifera. The presence of an archaeome in the terrestrial hosts varies substantially. With respect to abundant archaeal taxa, they harbor a higher proportion of methanoarchaea over the aquatic environment. We find that the archaeal community is much less diverse than its bacterial counterpart. Archaeal amplicon sequence variants are usually host-specific, suggesting adaptation through co-evolution with the host. While bacterial richness was higher in the aquatic than the terrestrial hosts, a significant difference in diversity and richness between these groups could not be observed in the archaeal dataset. Our data show a large proportion of unclassifiable archaeal taxa, highlighting the need for improved cultivation efforts and expanded databases.

## Graphical abstract

Relatedness of all model systems studied, and the five most abundant archaeal genera found in each system. Abundant genera are based on relative abundances in the amplicon data. Gray circles indicate the absence of detectable archaea. Red circles on the hosts identify sampled sites. Hosts are ordered using an unscaled phylogenetic tree based on species divergence times of a molecular time tree available through TimeTree5 and visualized in iTOL. Asterisks indicate branches where the tree is based on approximation through a close relative. The single *Cand*. Nitrosotalea or *Haloferaceae* found in *D. melanogaster and H. vulgaris*, respectively, were excluded as they could not be replicated. Where divergent from phylum names in the SILVA database, current names from the GTDB database were added.

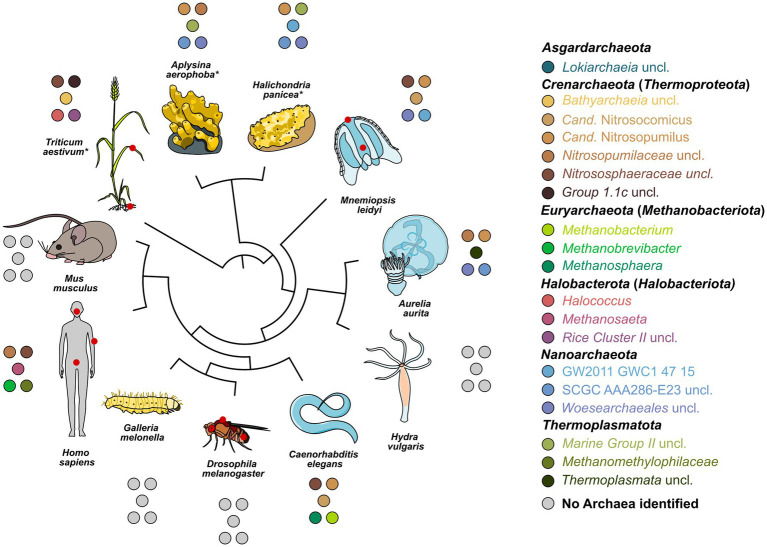

## 1 Introduction

The term metaorganism is used to describe the entity of any host organism together with its associated microbiota. Symbiotic interactions described within this context may include beneficial, neutral, and destructive relationships with the community (Bosch and McFall-Ngai, 2011; Tipton et al., 2019). While the bacterial community has been extensively studied in many multicellular host organisms, the archaeome, mycobiome, and virome remain less understood (Runge and Rosshart, 2021). This is partly because they comprise a smaller proportion of the overall microbiome, can be difficult to extract, or remain untargeted by sub-optimal primers (Bang and Schmitz, 2018). This results in an under-representation of archaea in both 16S rRNA gene amplicons and metagenomes.

Archaea can play an essential role in physiology and function of hosts ranging from plants to mammals (Taffner et al., 2018; Song et al., 2019; Youngblut et al., 2021). A considerable proportion of terrestrial microbiota, and particularly vertebrates, is comprised of methanoarchaea (Youngblut et al., 2021). They represent the last level in the trophic chain of the stepwise breakdown of organic matter in the host system, resulting in the production of methane (Borrel et al., 2020). Methanoarchaea have been found to be more prevalent in unhealthy humans with diseases, such as inflammatory bowel disease, periodontitis, or colon cancer (Borrel et al., 2020). There they may contribute to inflammation alongside fermentative bacteria. They have, however, never conclusively been identified as direct pathogens. In marine environments, archaea have predominantly been linked to nitrogen metabolism. Members of the *Crenarchaeota* are highly abundant in the water column (Karner et al., 2001), coral reefs (Rusch and Gaidos, 2013), and marine hosts within them (Bayer et al., 2008; Sharp et al., 2017), where they contribute substantially to ammonia oxidation.

In a previous study (Rausch et al., 2019), the bacterial community composition and diversity in animal metaorganisms were assessed in a similar setup of multicellular hosts with the aim to determine primer-specific differences of the composition in relation to the metagenome. The study included ten multicellular hosts spanning from marine invertebrates to insects, mammalian hosts, and plants. It identified the transition between aquatic and terrestrial hosts as a major event in microbiome evolution (Rausch et al., 2019). Here, we aim to further complete our understanding of the microbiome by investigating the archaeome composition and diversity in a small set of five to ten samples from selected metaorganisms using amplicon sequences of the 16S rRNA gene. These hosts represent model organisms and life stages studied within the Collaborative Research Centre “Origin and Function of Metaorganisms”^1^ in Kiel, Germany. Furthermore, we compare and combine our data with the corresponding bacterial communities extracted and amplified from the same host samples. As archaea often occur in syntrophic relationships with bacteria (Liu et al., 2018), we aim to establish to which extent we can detect these interaction partners through microbial network analysis. This study includes eleven multicellular hosts, namely, the Porifera *Aplysina aerophoba* and *Halichondria panicea,* the Ctenophora *Mnemiopsis leidyi,* the Cnidaria *Aurelia aurita* and *Hydra vulgaris,* the nematode *Caenorhabditis elegans*, the two arthropods *Drosophila melanogaster* and the greater wax moth *Galleria mellonella*, the two vertebrates *Mus musculus* and *Homo sapiens*, as well as the vascular plant *Triticum aestivum*. As amplicon primers were developed and benchmarked elsewhere (Pausan et al., 2019), we focus on the archaeal community composition and diversity. We expected to find similar patterns in archaeal diversity and richness, as were previously found in the bacterial community. This includes a higher diversity and richness in marine over terrestrial hosts, as was previously shown (Rausch et al., 2019). These data can provide a starting point for further research into cross-domain interactions, highlighting which hosts are particularly suited to this study.

## 2 Materials and methods

### 2.1 Host cultivation and DNA extraction

#### 2.1.1 *Aplysina aerophoba* and *Halichondria panicea* (*Porifera*)

*Aplysina aerophoba* individuals were collected in the Mediterranean close to L’Escala, Spain, in April and June 2019 by snorkeling (*n* = 9). *H. panicea* individuals (*n* = 10) were sampled by snorkeling in the Baltic Sea in the Kiel bight in November 2019 and June 2020. Sponges were transported submerged in seawater and in cooling units to the Institute of Marine Science (ICM-CSIC) in Barcelona, Spain, and to the GEOMAR Helmholtz Centre for Ocean Research Kiel, Germany, respectively. Both sponge species were maintained for 1 to 2 weeks in individual flow-through aquaria with direct intake of seawater that contained microplankton as a natural food source. Small pieces of tissue were dissected from *A. aerophoba* and *H. panicea* with sterile forceps and scalpels. The tissue was rinsed with sterile-filtered artificial seawater to remove loosely attached microorganisms, then preserved in RNAlater at 4°C for 24 h, and stored at-80°C. DNA was extracted from sponge samples with the DNeasy Power Soil Kit (Qiagen, Hilden, Germany), according to Busch et al. (2022). The two chosen sponge species differ in their bacterial abundance and diversity (Gloeckner et al., 2014; Moitinho-Silva et al., 2017). *Aplysina aerophoba* belongs to the so-called high microbial abundance (HMA) sponges that are characterized by a highly diverse and dense microbiome. *Halichondria panicea* is known as a low-microbial abundance (LMA) sponge that is dominated by a single bacterial symbiont and less dense microbiota (Knobloch et al., 2019).

#### 2.1.2 *Mnemiopsis leidyi* (*Ctenophora*)

*Mnemiopsis leidyi* (*n* = 5) were caught in the Kiel Fjord (geographic location 54.3312 N, 10.1499E), and DNA was extracted 4 h after the catch from epidermis and gut as described in Rausch et al. (2019). In short, the tissue of the ctenophores was washed with sterile artificial seawater and dissociated overnight using collagenase (Sigma-Aldrich, St. Louis, MO, United States). Prokaryotic and eukaryotic cells were separated through filtering through nylon gauze, the addition of IGEPAL CA-630 (Sigma-Aldrich) and centrifugation. DNA was subsequently extracted from the prokaryotic fraction using the Wizard genomic purification kit (Promega, Madison, WI, USA) and manufacturer’s instructions with additional incubations steps with EDTA, lysozyme, and Proteinase K.

#### 2.1.3 *Aurelia aurita* polyps and medusae (*Cnidaria*)

*Aurelia aurita* polyps of the subpopulation North Atlantic (Roscoff) were kept at 20°C in artificial seawater (ASW; 30 practical salinity units (PSU); Tropical Marine Salts, Tropic Marin), in 2 L plastic tanks. Polyps were fed twice a week with freshly hatched *Artemia salina* (HOBBY, Grafschaft-Gelsdorf, Germany) and washed weekly with ASW. Strobilation, a key reproductive process in the Scyphozoan life cycle, was induced with 5 μM 5-methoxy-2-methyl indole (Carl Roth, Karlsruhe, Germany) for 3 days, after which the inducer was omitted. During strobilation, the polyp undergoes a series of morphological changes, including segmentation and finally the release of disk-shaped ephyrae. Ephyrae represent the next stage in the *Aurelia* life cycle and are essentially miniature medusae. Once liberated from the strobila, they exist as distinct, free-swimming entities (Spangenberg, 1965). The release of ephyrae was observed from day 12 onwards. Ephyrae were transferred into a 30 L Kreisel aquarium (Quallenwelt, Czech Republic), fed daily, and washed weekly. Ephyrae developed into mature medusae of 8 cm diameter. Preceding DNA extraction, polyps and medusae were not fed for at least 3 days to ensure empty guts.

Polyps (*n* = 10) were washed three times with sterile ASW. Polyps were mixed with 480 μL of 50 mM EDTA and homogenized with a motorized pestle (Kontes, DWK Life Science, Wertheim, Germany). Young medusae (*n* = 8) were taken from the aquarium, washed three times with sterile ASW, and frozen with liquid nitrogen for storage at −80°*C. Prior* to DNA extraction, medusae were thawed for 2 h at 37°C and homogenized in a mixer. The homogenate was centrifuged for 30 min at 4°C and 4,000 rpm. The pellet was resuspended in 500 μL 50 mM EDTA, and the cells were mechanically disrupted using a Geno/Grinder 2000 (BT&C/OPS Diagnostics, Bridgewater, NJ) with glass beads (2.7 mm, 1 mm, 0.1 mm; Carl Roth) for 3 min at 1,300 rpm. All samples were incubated for 30 min at 37°C with 120 μL 10 mg/mL lysozyme (Carl Roth, Karlsruhe, Germany) and 20 μL Proteinase K (3 Units, Thermo Fisher Scientific, Darmstadt, Germany). DNA extraction was performed using the Wizard genomic purification kit (Promega, Madison, WI, United States) according to the manufacturer’s instructions.

#### 2.1.4 *Hydra vulgaris* AEP (*Cnidaria*)

*Hydra vulgaris* AEP (*n* = 10) were maintained at 18°C following standard protocols (Lenhoff and Brown, 1970) and fed three times per week with newly hatched *Artemia salina* nauplii. Total DNA from five fresh *H. vulgaris* polyps was extracted using a ZymoBIOMICS DNA Microprep Kit (D4301, Zymo Research) following the manufacturer’s standard protocol. To achieve optimal lysis efficiency, a Precellys 24 (Bertin Technologies) homogenizer (5,000 rpm with three cycles of 60 s and 10 s pause in between) was used in the bead beating step.

#### 2.1.5 *Caenorhabditis elegans* (*Nematoda*)

*Caenorhabditis elegans* were isolated from compost (*n* = 5) and slug feces (*n* = 5). Compost and slugs were collected in August 2021 from the botanical garden in Kiel, Germany. Slug feces were collected within 24 h after sampling. The samples were placed individually in petri dishes, covered with sterile M9-buffer including 0.025% TritonX-100, and nematodes found were pipetted individually into 96 well plates containing DreamTaq buffer (Thermo Fisher Scientific, Waltham, MA, United States), Proteinase K (20 mg/mL, Thermo Fisher Scientific, Waltham, MA, USA), and two to four sterile 1-mm zirconium beads. The isolated worms were frozen at −80°C for 16 h, ground twice using a Geno/Grinder 2000 (SPEX SamplePrep, Metuchen, United States) for 3 min at 1500 strokes per minute, followed by boiling in a Thermo Cycler (SensoQuest GmbH, Göttingen, Germany) for 1 h at 50°C and 15 min at 95°C. Individual *C. elegans* were identified by PCR using the species-specific primers nlp30-F (ACACATACAACTGATCACTCA) and nlp30-R (TACTTTCCCCATCCGTATC) (Petersen et al., 2014). The cycling profile consisted of an initial denaturation for 2 min at 95°C, 35 cycles of 1 min at 95°C, 30 s at 55°C, and 1 min at 72°C, followed by 10 min at 72°C final extension. DNA of individual *C. elegans*-positive samples from the same compost or slug feces sample was joined and used for the subsequent analysis.

#### 2.1.6 *Drosophila melanogaster* (*Arthropoda*)

Wild-type flies (*w^1118^*) were raised at 25°C. Genomic DNA from whole flies (*n* = 5) and dissected intestines (*n* = 5) were extracted using the DNeasy Blood and Tissue Kit (Qiagen, Germany). The tissue was homogenized in 500 μL sterile PBS using a bead ruptor. After centrifugation at 4°C and 10,000 × g for 10 min, the pellet was resuspended in 180 μL enzymatic lysis buffer (20 mM Tris–HCl pH 8.0, 2 mM sodium EDTA, 1.2% Triton X-100 and 20 mg/mL lysozyme) and incubated for 45 min at 37°C. Next, 200 µ AL buffer and 25 μL Proteinase K were added, and samples were incubated at 56°C for 30 min. After adding 200 µL of 70% ethanol, samples were transferred to DNeasy Mini spin columns and centrifuged at 10,000 × g for 1 min. Then, samples were washed first with 700 μL AW1 buffer and centrifuged for 1 min at 10,000 × g and then with 500 μL AW2 buffer and centrifuged for 3 min at 20,000 × g. Samples were eluted with 50 μL AE buffer. A negative control was prepared using sterile PBS treated the same way as samples.

#### 2.1.7 *Galleria mellonella* (*Arthropoda*)

*Galleria mellonella* last instar larvae were purchased from Faunatopics Gmbh (Marbach, Germany). Upon arrival, larvae were stored at 10°C for a maximum of 2 weeks without feeding. Healthy, motile larvae with a body weight of 350–450 mg and without any signs of melanization and diarrhea were selected for the experiment (*n* = 10, one larva per a Petri dish). Upon overnight acclimatization at room temperature, fecal pellets (one or two per insect) were collected from each larva and stored at −80°C. Larvae were then surface-sterilized by submerging into 70% ethanol and aseptically dissected. The distal sections of larval intestines were isolated, placed into a sterile 1.5 mL Eppendorf tube, and immediately snap-frozen in liquid nitrogen. All samples were delivered on the same day on dry ice for DNA extraction, which was done as described for *Homo sapiens* below.

#### 2.1.8 *Homo sapiens* (*Chordata*)

Healthy donors were recruited from a blood donor cohort (incl. health criteria that apply for blood donation) in North Germany (Schleswig-Holstein) and randomly selected for this study. The cohort had a mean age of 56, consisted of 70% females with a mean BMI of 26. Donors were asked to provide stool, buccal swab, and skin samples for microbiome analysis. Stool samples were collected in tubes without stabilizer, buccal swab samples were sampled from the inside of the cheek by rubbing the cotton head with a little pressure 10–15 times, and skin samples were taken from the crook of the arm to examine the skin microbiome. After transporting the samples to the Microbiome laboratory of the Institute of Clinical Molecular Biology, samples were stored at −80°C until further processing. The study was conducted in accordance with the Declaration of Helsinki, and ethics approval was granted by the ethics committee at Kiel University (AZ A103/14). DNA of stool samples (*n* = 10) was extracted using the QIAamp DNA fast stool mini kit automated on the QIAcube (Qiagen, Hilden, Germany). The material was transferred to 0.70 mm Garnet Bead tubes (Dianova, Hamburg, Germany) filled with 1.1 mL InhibitEx lysis buffer. For oral (buccal swabs, *n* = 10) and skin (swab, *n* = 10) samples, QIAamp UCP Pathogen mini kit automated on the QIAcube was used. The swab was transferred to a Pathogen Lysis Tube S filled with 0.65 mL ATL buffer (incl. DX) and incubated for 10 min at 56°C with continuous shaking at 600 rpm. Bead beating for both sample types was performed using a SpeedMill PLUS (Analytik Jena, Jena, Germany) for 45 s at 50 Hz with subsequent continuation of the manufacturer’s protocol. The extracted DNA was stored at −20°C prior to PCR amplification. Blank extraction controls were included during extraction of samples.

#### 2.1.9 *Mus musculus musculus* and *Mus musculus domesticus* (*Chordata*)

The mouse subspecies *M. m. musculus* (*n* = 5) and *M. m. domesticus* (*n* = 5) were raised in captivity, euthanized, and their caecum dissected for DNA extraction with the DNA/RNA AllPrep kit (Qiagen) and a bead beating step using Lysis Matrix E (MP Biomedicals, Irvine, CA, United States) as described in Rausch et al. (2019).

#### 2.1.10 *Triticum aestivum* (*Tracheophyta*)

Wheat cultivation and DNA extraction from leaves (*n* = 10) and roots (*n* = 10) were done as described in Seybold et al. (2020). In brief, wheat seedlings of the cultivar Obelisk were propagated under standardized conditions (16-h light/8-h dark cycles at 15°C) in a climate chamber (Percival plant growth chambers, CLF PlantClimatics GmbH, Wertingen, Germany). From 2-week old leaf seedlings, the second leaves were harvested and used for DNA extraction as previously described (Hassani et al., 2020; Seybold et al., 2020). In brief, cells were homogenized in a bead beating step using Lysis Matrix E (MP Biomedicals), followed by a lysozyme, Proteinase K, and RNase A treatment. DNA extraction was then carried out using the FastDNA SPIN kit for soil (MP Biomedicals) according to the manufacturers’ instructions.

### 2.2 Amplification and sequencing of archaeal and bacterial 16S rRNA genes

For bacterial amplicons, the V3–V4 variable regions of the 16S rRNA gene were amplified in a one-step PCR as described by Rausch et al. (2019) using the primer pair 341F-806R (5′-CCTACGGGAGG-CAGCAG-30 and 5′-GGACTACHVGGGTWTCTAAT-30) (Kozich et al., 2013). After verification of the presence of PCR products by gel electrophoresis, normalization (Sequal Prep Normalization Plate Kit; Thermo Fisher Scientific, Waltham, United States) and pooling were performed.

For sequencing of archaeal 16S rRNA genes, a nested-PCR approach was chosen using the PCR reactions and cycling conditions described by Pausan et al. (2019). In brief, a first round of PCR was performed using primer pair 344F-1041R (25 cycles), followed by a second round using the universal primer pair 519F-806R (30 cycles). In-between, PCR products were purified using the MinElute PCR Purification kit (Qiagen; Hilden, Germany). Normalization of final PCR products was done using the Sequal Prep Normalization Plate Kit (Thermo Fisher Scientific, Waltham, United States), and pooling was performed. Archaeal and bacterial amplicon sequencing was conducted on a MiSeq platform (MiSeq; Illumina, San Diego, United States) with v3 chemistry. The settings for demultiplexing were 0 mismatches in the barcode sequences.

### 2.3 Bioinformatics sequence processing and taxonomic annotation

Amplicon sequence data were processed using the workflows provided by Rühlemann, M. on GitHub at https://github.com/mruehlemann/ikmb_amplicon_processing (Accessed: 17.01.2024). In short, processing included primer clipping using cutadapt 4.1 (Martin, 2011), followed by quality filtering and trimming with the DADA2 package (version 1.42) (Callahan et al., 2016) and the filterAndTrim()-function with parameters truncLen = c (265, 245), maxN = 0, maxEE = c (2, 2), truncQ = 5, rm.phix = TRUE. All sequences were dereplicated, clustered into bacterial or archaeal amplicon sequence variants (ASVs), and merged using DADA2. Each run was processed individually and then merged. After merging of the sequence tables, chimeras were removed using DADA2, and taxonomy was assigned using the Bayesian classifier and the SILVA database v138.1 NR99 (Quast et al., 2013).

### 2.4 Data analysis and visualization

For ease of interpretation, bacterial amplicon sequence variants were renamed to BSVs and will be referred to as such in the following, while archaeal amplicon sequence variants remain ASVs. All data analyses were done using Rstudio V2022.07.01 (RStudio Team, 2023) and R 4.3.2 (R Core Team, 2023). The data were transformed and managed using the phyloseq (McMurdie and Holmes, 2013), microViz (Barnett et al., 2021), and ampvis2 packages (Andersen et al., 2018). ASV and BSV tables were decontaminated by first filtering out all extrinsic and unwanted domains, including chloroplasts, mitochondria, eukaryotes, and bacteria or archaea, respectively (Supplementary Figure S1). They were then statistically decontaminated using decontam (Davis et al., 2018) with a prevalence threshold of 0.5 and all negative controls (prep and sequencing controls) as decontamination reference. Identified contaminants (Supplementary Figures S2, S3) were removed from the dataset, as well as samples with less than 500 reads. In the case of the archaeal dataset, 18 remaining ASVs which occurred in negative controls were removed in addition. Data were transformed into relative abundances for heatmaps of the most abundant genera. Principal coordinates analysis was plotted based on Bray–Curtis dissimilarity matrices of all untransformed ASVs and BSVs with an abundance of 0.01% in at least one sample. Whether samples clustered distinctly by host, sample type or environment (aquatic/terrestrial) was assessed using PERMANOVA and pairwise PERMANOVA with 999 permutations and the packages vegan (Oksanen et al., 2020) and pairwiseAdonis (Martinez Arbizu, 2020). Diversity and richness indices were calculated based on ASV/BSV tables rarefied to 10,000 reads, which covered both domains well (Supplementary Figures S4–S6). Differences in diversity means between terrestrial (*C. elegans*, *D. melanogaster*, *G. melonella*, *H. sapiens*, *M. musculus*, and *T. aestivum*) and aquatic (*A. aurita*, *A. aerophoba*, *H. panicea*, *H. vulgaris,* and *M. leidyi*) hosts were calculated using Kruskal–Wallis test with a post-hoc Wilcoxon test. The phylogenetic autocorrelation index Moran’s *I* (Gittleman and Kot, 1990) was calculated using the R package ape (Paradis and Schliep, 2019). This included the Shannon *H′* diversity index and Chao1 richness index of the bacterial and archaeal community, respectively, against species divergence times of a molecular time tree available through TimeTree5 (Kumar et al., 2022). Three hosts were not directly available and had to be substituted with their closest available relatives, namely, *H. panicea* replaced with *Superitida*, *A. aerophoba* replaced with *Aplysina fulva,* and *T. aestivum* replaced with *Hordeum vulgare*. Hosts with no detectable archaea community were dropped from the tree for the analysis of Moran’s *I* and the archaeal diversity and richness indices. The molecular time tree including all hosts was visualized in iTOL (Letunic and Bork, 2021) for use in the graphical abstract. Bacterial and archaeal indicator analysis for the marine hosts *A. aerophoba*, *H. panicea*, *A. aurita,* and *M. leidyi* was done using the indicspecies package (De Cáceres and Legendre, 2009). Associations between sequence variants with an abundance >0.1% and host organisms were calculated using a multilevel pattern analysis (multipatt) with the “r.g” function and 999 permutations to determine significance. All indicators from the multipatt analysis with a *p* < 0.05 were plotted in Cytoscape (3.10.1). Association networks of archaea, bacteria, and archaea with bacteria were calculated using SpiecEasi (Kurtz et al., 2015) with Meinshausen-Buhlmann’s neighborhood selection (mb), a lambda of 100, lambda min ratio of 0.001, and 100 replications of the Bounded StARS method. To reduce the sparsity of the data, only sequence variants that occur with at least 3 reads in 10% of the dataset were considered. To estimate robustness of the analysis, 100 random networks (Erdős-Rényi random graphs) were calculated using the same number of nodes and edges or clustering coefficient of each network (Supplementary Figure S7). The random networks and the community-based networks with shared nodes and edges showed the same degree distributions, which resembled a Poisson distribution across 12 degrees (Supplementary Figure S7). Network characteristics were evaluated using the NetCoMi package (Peschel et al., 2021), as well as basic statistics from igraph (Csárdi and Nepusz, 2006), including degree distribution, diameter and radius, betweenness, connectedness (eigenvectors), and the calculation of hubs.

### 2.5 Ethics statement and permissions

Mice were maintained and handled according to FELASA guidelines and German animal welfare law (Tierschutzgesetz § 11, permit from Veterinäramt Kreis Plön: 1401–144/PLÖ–004697). The study was conducted in accordance with the Declaration of Helsinki, and ethics approval was granted by the ethics committee at Kiel University for human samples (AZ A103/14).

## 3 Results

### 3.1 Sample processing and quality control

The archaeal dataset started out with a total of 5,871,779 processed reads and 8,775 ASVs, of which 2,805,728 reads and 2,783 ASVs remained after decontamination and removal of Bacteria, Eukarya, mitochondria, and chloroplasts. The bacterial dataset started out with 6,045,133 reads and 20,113 BSVs. After decontamination and removal of unwanted domains, 5,190,629 reads and 19,386 BSVs remained in the dataset. A detailed overview of overall and per host reads and number of sequence variants can be found in Supplementary Table S1. Identified contaminants include human-associated *Methanosphaera* and *Methanobrevibacter*, as well as *Nitrososphaeraceae* in the marine hosts (Supplementary Figure S3A). As archaeal positive controls are based on human *Methanosphaera* isolates and cannot be distinguished from the real data, this results in strict removal of *Methanosphaera* ASVs from human samples. Non-human-associated *Methanosphaera* remain in the dataset. Some of the identified bacterial contaminants include individual BSVs from host-specific symbionts (e.g., *Amylibacter* or *Synechococcus*). Where known these were added back into the dataset (Supplementary Figure S3B).

### 3.2 Comparison of archaeal community composition and diversity among animal host

**Figure 1:**
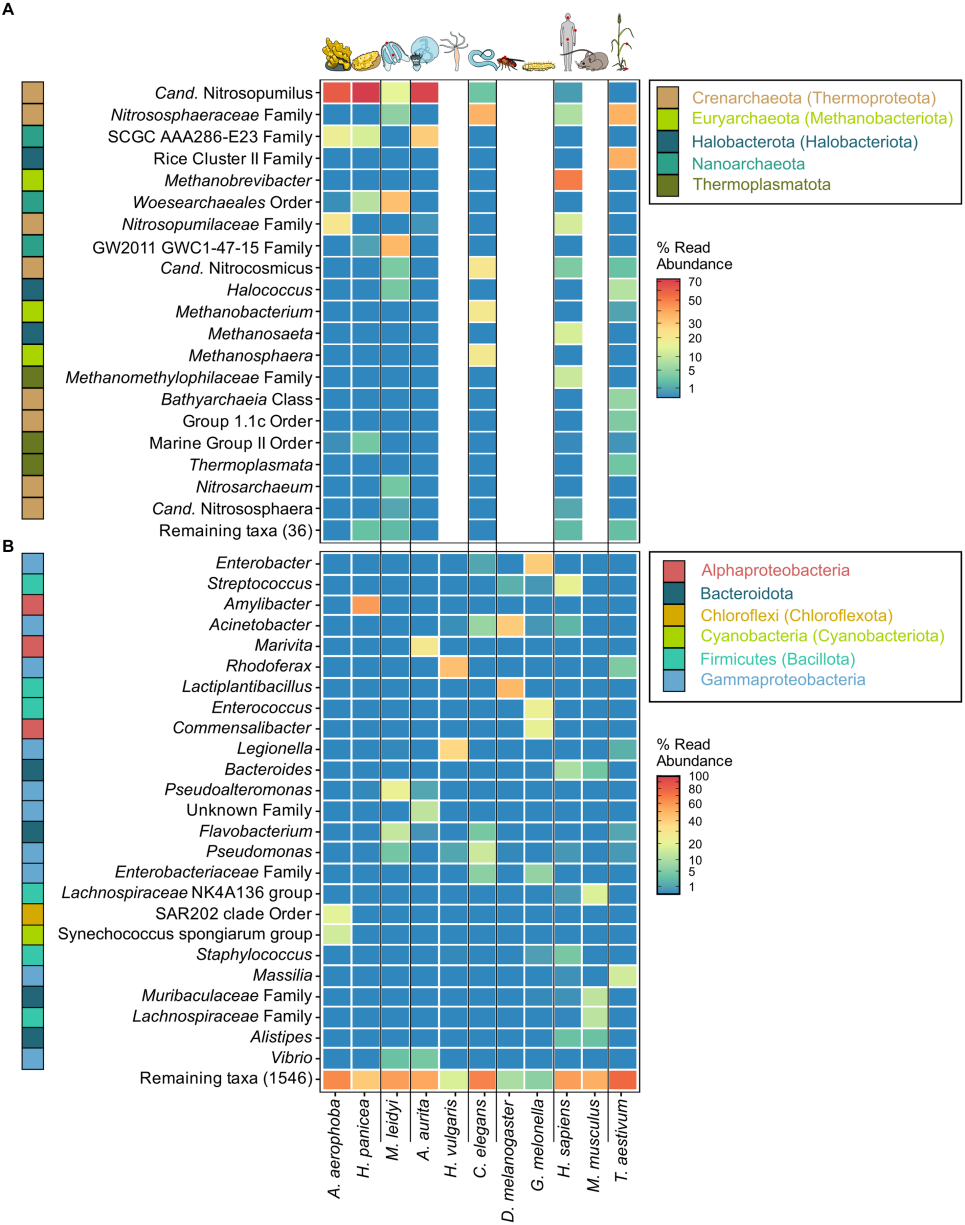
Most abundant archaeal **(A)** and bacterial **(B)** genera across the tested host organisms. The heatmaps are based on relative abundances where taxa with an abundance <0.01 are plotted in the same shade of blue. The best possible taxonomic assignment was used to label each group. Phylum affiliations of each genus are indicated through colored blocks to the left of the genus names. Phyla include names according to the GTDB taxonomy where divergent from the SILVA taxonomy. *Proteobacteria* (*Pseudomonadota*) are plotted as *Alpha- and Gammaproteobacteria* on the class level.

**Figure 2:**
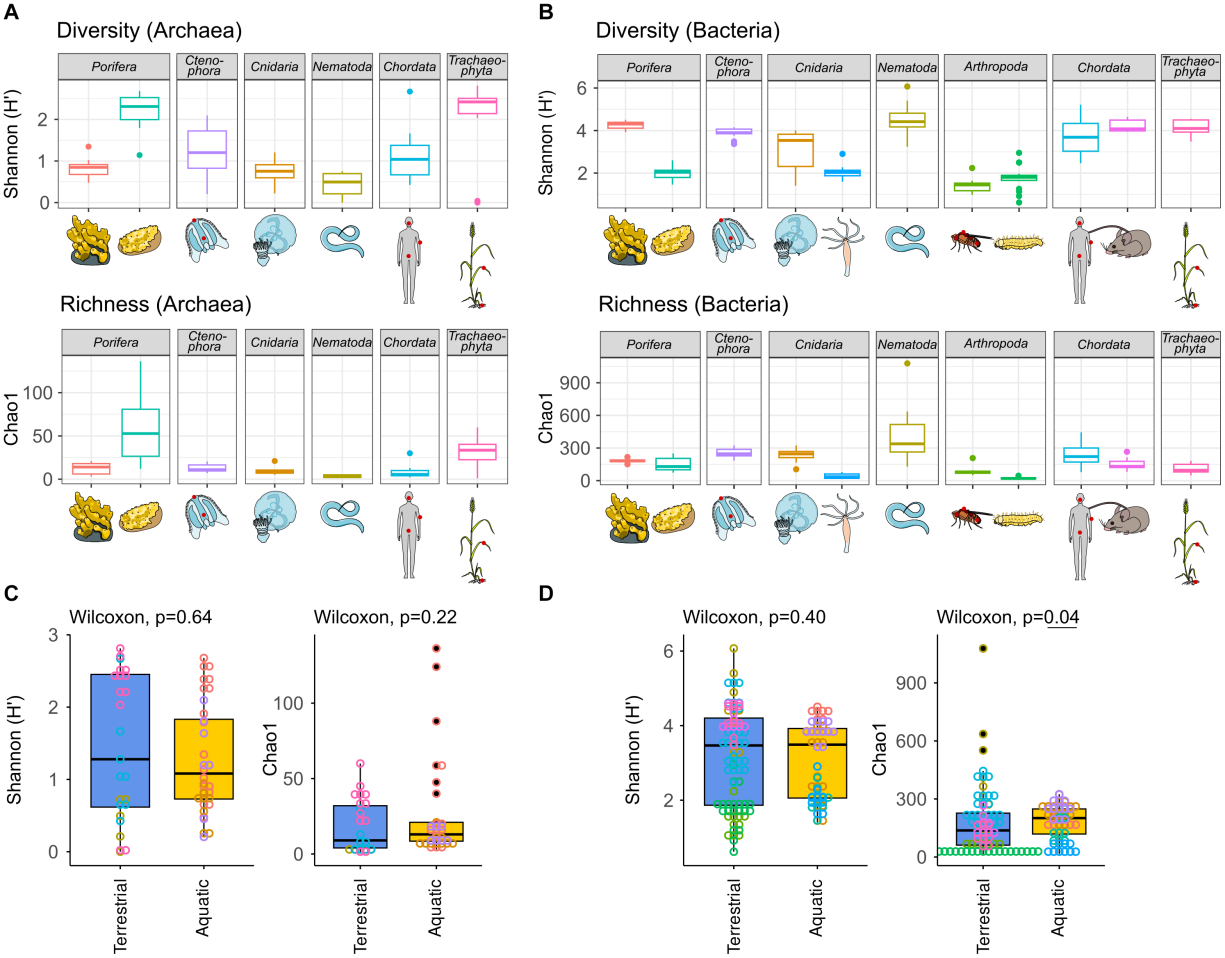
Archaeal **(A)** and bacterial **(B)** diversity and richness indices, as well as group comparisons between aquatic and terrestrial hosts **(C,D)**. Diversity (*Shannon H′*) and richness (Chao1) indices were calculated based on all samples rarefied to 10,000 reads. Hosts were grouped as terrestrial [*H. sapiens*, *M. musculus*, *C. elegans*, *D. melanogaster*, *G. melonella*, and *T. aestivum*] or aquatic [*A. aerophoba*, *H. panicea*, *A. aurita*, *M. leidyi*, and *Hydra vulgaris*]. Differences in diversity and richness means between the groups were tested using a Kruskal–Wallis test with a significance cutoff <0.05 for both the archaeal **(C)** and the bacterial **(D)** communities.

The archaeome of all host organisms is captured by the most abundant 25 archaea (Figure 1A), which belong to the five archaeal phyla *Crenarchaeota*, *Euryarchaeota*, *Halobacterota*, *Nanoarchaeota,* and *Thermoplasmatota*. Marine Porifera, Ctenophora, and Cnidaria harbor mainly *Cren*- and *Nanoarchaeota*, including *Nitrosopumilus* with >60% relative abundance. The low-microbial abundance (LMA) sponge *H. panicea* harbored a high archaeal diversity and richness in comparison with the other marine hosts and particularly the high microbial abundance (HMA) sponge *A. aerophoba* (n = 9). Comparison of diversity and richness means using the Kruskal–Wallis test on aquatic and terrestrial hosts showed no significant difference between the groups (Shannon *H′*: χ^2^ = 0.22, value of *p* = 0.6389; Chao1: χ^2^ = 1.4843, value of *p* = 0.2231, Figure 2C). *Caenorhabditis elegans* (*n* = 5) host uncultured *Nitrososphaeraceae*, *Cand.* Nitrocosmicus, as well as *Methanobacterium* and *Methanosphaera*, all at 20–40% relative abundance. Humans harbor predominantly *Methanobrevibacter* and to a lesser extent *Methanomethylophilaceae* located in the gut (Figure 1A; Supplementary Figure S5). Skin samples harbor unclassified *Crenarchaeota,* while oral samples showed the genus *Methanosaeta* (Figure 1A; Supplementary Figure S5). *Triticum aestivum* as only plant representative hosts *Halobacterota*, including members of the Rice Cluster II family and *Halococcus*, but also unclassified *Nitrososphaeraceae* (Figure 1A). The diversity and richness among terrestrial hosts are highest in the roots of *T. aestivum*. No single archaea could be detected in the insect hosts *G. mellonella*, as well as in the mouse subspecies *M. musculus musculus* and *M. m. domesticus.* In *D. melanogaster* and *H. vulgaris,* five ASVs remained in one sample each belonging to either *Cand.* Nitrosotalea or *Haloferaceae*. These, however, were excluded from the dataset as they could not be replicated. We therefore consider them to lack an archaeal community.

### 3.3 Community composition and diversity of host-associated bacteria

**Figure 3:**
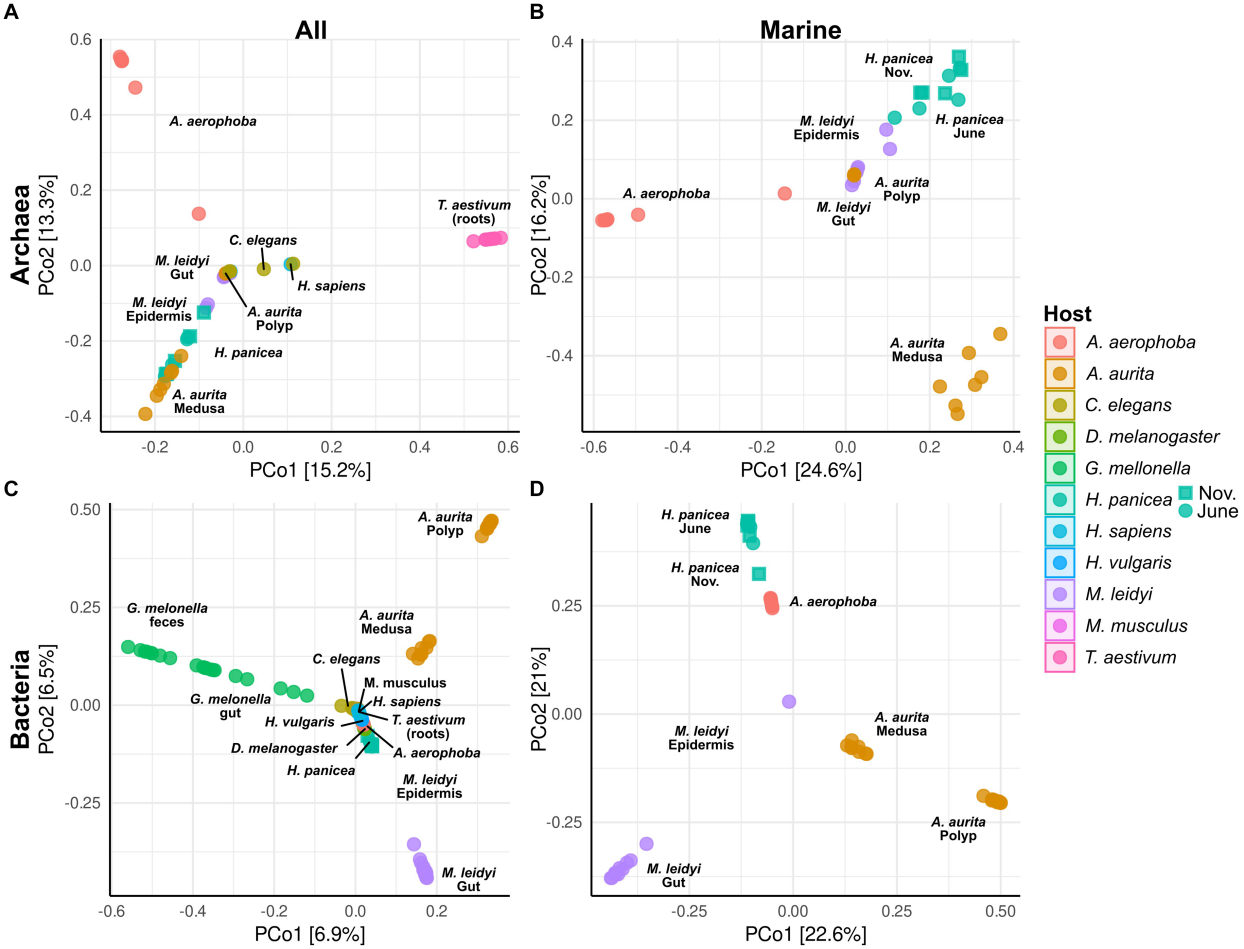
Principal coordinate analysis (PCoA) based on Bray–Curtiss dissimilarity of the archaeal **(A,B)** and bacterial **(C,D)** communities. Plots A and C are based on all hosts, while plots B and D show only the marine hosts (*A. aerophoba*, *A. aurita*, *H. panicea*, and *M. leidyi*). For the different *H. panicea* subsamples (seasons), these have been plotted either as circle (June) or as square (November). Only ASVs and BSVs with an abundance of >0.01% in at least one sample were considered. No initial data transformation was applied. The relative contribution (eigenvalue) of each axis to the total inertia in the data is indicated as percentage in the axis titles.

The bacterial host-associated community from the same samples is more diverse than the archaeal community (Figure 2) resulting in only one to five abundant taxa (≥1%) per host among the top 25 bacterial genera and a high proportion of remaining taxa (Figure 1B). The sponge *A. aerophoba* hosts *Chloroflexi* of the SAR202 clade, as well as the *Cand.* Synechococcus spongiarum group, while *H. panicea* shows a member of the genus *Amylibacter* with a relative abundance above 60%. The number of taxa is reflected in the bacterial diversity, which is higher in *A. aerophoba* than in *H. panicea*. *M. leidyi*’s main colonizers are *Pseudoalteromonas* and *Flavobacterium* (Figure 1B). *A. aurita* harbors the genus *Marivita* at 30–40% and an unclassified *Gammaproteobacterium* at 20%. Differences in *A. aurita* community composition can be attributed to the polyp and medusa life stages (Figure 3; Supplementary Figure S4). While the unclassified *Gammaproteobacterium* is most abundant in the polyp stage, together with *Vibrio* and different *Rhodobacteraceae*, *Marivita* is the most abundant genus on the *A. aurita* medusae, alongside *Marinobacter.* The most abundant genus shared between the life stages is *Formosa*. The *H. vulgaris* bacterial community consists of the genera *Rhodoferax* and *Legionella* with less than 20% of other genera. *Curvibacter* which are often present at relative abundances >70% in *H. vulgaris* (Franzenburg et al., 2013; Taubenheim et al., 2022) were not observed here. This may potentially be linked to the primer sets used in the studies. While here we use the bacterial V3–V4 region, the previously mentioned studies use the V1–V2 region for their amplicon analysis. Further differences may arise due to a change in the extraction kit. As here the ZymoBIOMICS DNA Microprep Kit was used instead of a chloroform/isoamyl alcohol extraction to optimize for the extraction of archaea. *Pseudomonas* contribute up to 20% of the *C. elegans* bacterial community. Other genera reach a maximum of 10%, including, for instance, *Acinetobacter* and *Enterobacteriaceae* (Figure 1B). *C. elegans* has the highest bacterial diversity and richness among the studied hosts (Figure 2B). Insect hosts have the lowest bacterial diversity and richness (Figure 2B). While *D. melanogaster* hosts mainly *Acinetobacter* and *Lactiplantibacillus*, *G. mellonella* harbor *Enterobacter, Enterococcus,* and *Commensalibacter.* The most abundant bacterial taxa in the human dataset each represent a particular subsample (Supplementary Figure S5). The oral dataset harbors the bacteria *Streptococcus*, *Veillonella,* and *Haemophilus*, while *Staphylococcus* and *Cutibacterium* are particularly abundant on skin. The stool samples contain the most diverse human-associated community including *Bacteroides*, *Alistipes*, and *Escherichia-Shigella* (Figure 1B; Supplementary Figure S5). While mice also host *Bacteroides* in their guts, their most abundant genera are made up of different *Lachnospiraceae* and *Muribaculaceae.* Wheat, in this case the roots, have a heterogenous and diverse bacterial community where the most abundant taxa are present at comparatively low relative abundances (Figures 1B, 2). The genus *Massilia* represents the most abundant genus at approximately 20% (Figure 1B). Overall, the bacterial diversity (Shannon *H′*) between terrestrial and aquatic hosts does not differ significantly (χ^2^ = 0.71, *p* = 0.40). Bacterial richness (Chao1) is significantly higher among the aquatic hosts (χ^2^ = 4.22, *p* = 0.04, Figure 2B), matching the pattern observed by Rausch et al. (2019).

### 3.4 Bacterial and archaeal community structure across hosts and host environments

Principal coordinate analyses on the archaeal and bacterial community composition were done using Bray–Curtis dissimilarity matrices and all taxa with an abundance higher than 0.01% in at least one sample (Figure 3). All host-specific communities differ significantly from one another except for *C. elegans* and *H. sapiens* in the archaeal community (Supplementary Table S2). Significant within-host differences among the marine hosts can be observed between the polyp and medusa life stages of *A. aurita*, both in the bacterial (*p* < 0.001) and archaeal community (*p* < 0.01, Supplementary Table S3). The community of *H. panicea* shows slight seasonal differences in its bacterial community composition (*p* = 0.02) but not in the archaeal community (*p* = 0.14). Comparing terrestrial with aquatic hosts, PERMANOVA indicates that communities differ significantly from one another (Archaea: *p* = 0.001, Bacteria: *p* = 0.001).

In addition, an approximate time tree of the closest available hosts was used to test whether phylogenetic autocorrelation (Moran’s *I*) may influence bacterial and archaeal species diversity and richness. Regarding the bacterial community, Moran’s *I* calculated with the diversity (Shannon *H′*) and richness (Chao1) of all hosts determined a positive autocorrelation with diversity (*p* = 0.02, SD = 0.08) but not richness (*p* = 0.20, SD = 0.07). A closer look at the phylogenetic autocorrelation within terrestrial and aquatic hosts showed that neither aquatic hosts (Shannon *H′*: *p* = 0.59, SD = 0.04; Chao1: *p* = 0.15, SD = 0.03) nor terrestrial hosts (Shannon *H′*: *p* = 0.18, SD = 0.21; Chao1: *p* = 0.20, SD = 0.17) exhibited a significant Moran’s *I*. Regarding the archaeal community, there was no phylogenetic autocorrelation across hosts for Shannon *H′* (*p* = 0.37, SD = 0.02) or Chao1 (*p* = 0.66, SD = 0.03). Notably, the four hosts without detectable archaeal community, namely, *H. vulgaris*, *D. melanogaster*, *G. mellonella,* and *M. musculus*, were excluded from this analysis. Consequently, Moran’s *I* could not be calculated for the terrestrial hosts as insufficient data were available. The archaeal community among the aquatic hosts also showed no significant autocorrelation with diversity or richness (Shannon *H′*: *p* = 0.14, SD = 0.02; Chao1: *p* = 0.26, SD = 0.01).

### 3.5 Archaeal and bacterial indicator taxa for marine hosts

**Figure 4:**
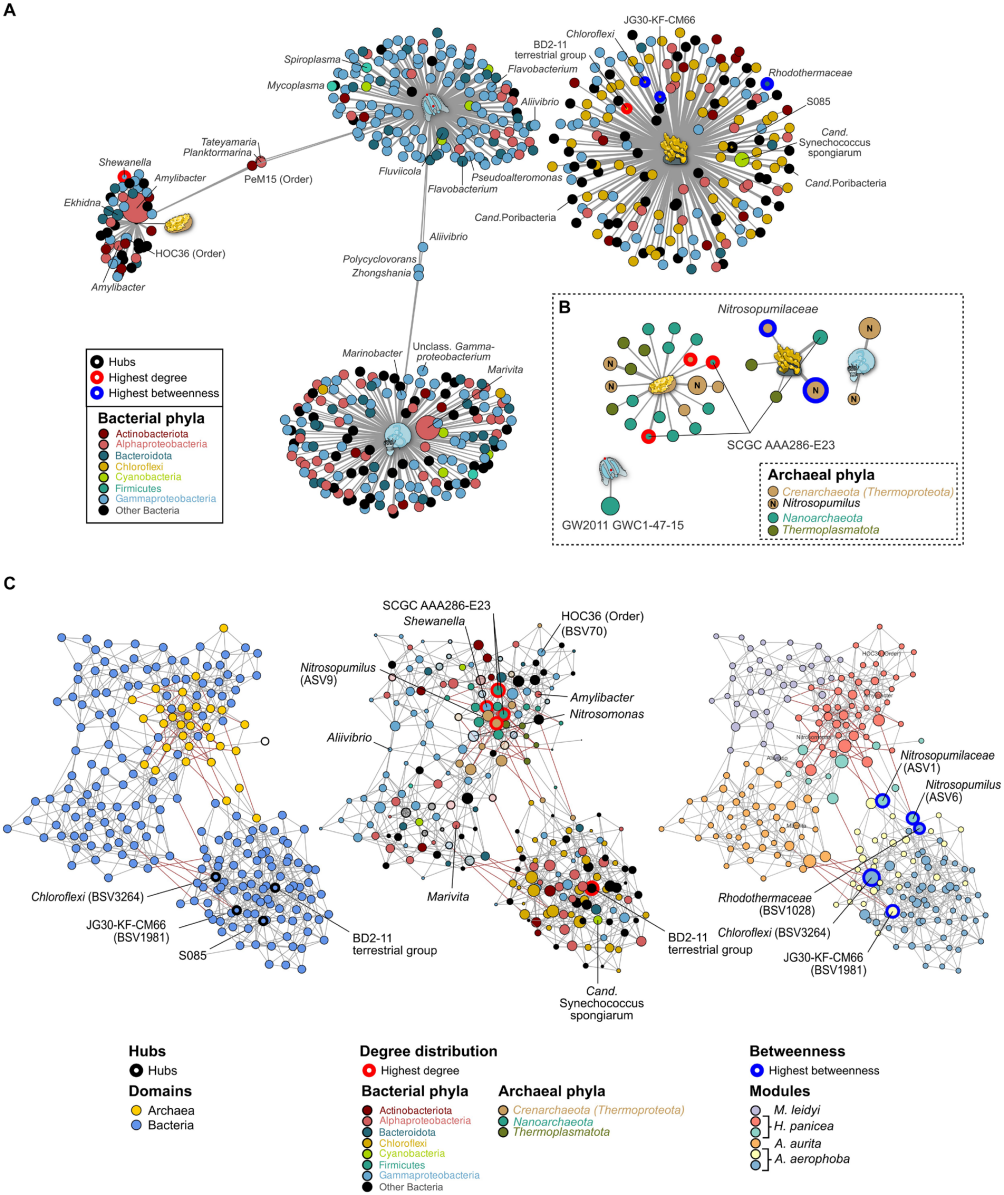
Indicator **(A,B)** and association network analysis **(C)** on the bacterial and archaeal community in the marine hosts. **(A)** Bacterial and **(B)** archaeal indicators (>0.1%) for each host are colored by phylum. GTDB taxonomy is added where divergent from the SILVA database. Taxa with an abundance >1% are plotted by size relative to their average abundance in the dataset. Edge lengths indicate the association strength to the target, where larger distances are less strongly associated. **(C)** Association networks calculated using both the bacterial and archaeal communities and plotted using three different main aspects. Left: Network highlighting the bacterial (blue) and archaeal (yellow) domain. Middle: The same network but with node sizes adjusted to the number of degrees (connections) to other taxa. The most connected nodes are highlighted in red. Nodes are colored to reflect their phylum affiliation. All taxa which do not occur as indicator taxa are opaque. Right: Nodes are colored according to the six modules identified through NetCoMi analysis and correspond roughly to each host. Size of the bubbles is scaled according to their betweenness values. Nodes with the highest betweenness are highlighted by blue circles. Known symbionts, abundant taxa, and key species from the association networks are highlighted by name.

Indicator taxa for the different marine hosts were calculated using the point-biserial indicator value. In the case of the bacterial community, *Gammaproteobacteria* and *Alphaproteobacteria* are associated with *A. aurita* and *M. leidyi* with similar association strength to each host. Overall, more *Alphaproteobacteria* occur on *A. aurita* (*n* = 36) than on *M. leidy* (*n* = 16)(Figure 4A). This includes *Marivita*, which is closely associated with *A. aurita* and particularly its medusa stage. *M. leidyi* is further associated with two *Firmicutes* belonging to the genera *Mycoplasma* and *Spiroplasma.* Only three indicator taxa of the *Gammaproteobacteria*, including *Aliivibrio*, are shared between *M. leidyi* and *A. aurita,* highlighting that their communities are largely distinct. The same applies to the archaeal community, where no indicators were shared among all the marine hosts. While *M. leidyi* is associated with a member of the *Nanoarchaeota, A. aurita* is connected solely to *Nitrosopumilus* ASVs. The number of associated archaeal phyla is higher in *H. panicea* and *A. aerophoba*, where we find four and two *Thermoplamatota* indicators, respectively. Both are preferentially colonized by abundant *Nitrosopumilus,* however, separate ASVs, including the highly abundant ASV9 (*Nitrosopumilus*). Bacterial indicators are highly distinct between the two porifera and mirror the trend of HMA versus LMA we find in the bacterial diversity. Archaeal indicators also reflect the higher diversity we find among *H. panicea*. In numbers we find that *H. panicea* is associated with 50 BSVs and 22 ASVs, while in *A. aerophoba* we find 184 BSVs and 5 ASVs to be indicator taxa (Figure 4). *Halichondria panicea* resembles *A. aurita* and *M. leidyi* more closely regarding phylum affiliation and shares three indicators, two *Alphaproteobacteria* and one *Actinobacteriota* BSV, with *M. leidyi*. The association strength of BSVs to the sponge host is, however, stronger than in the ctenophore and cnidarian. Its archaeal indicators are ASVs, belonging to the *Nanoarchaeota* and the *Nitrosopumilus* genus. *A. aerophoba* does not share any indicators with the other hosts and has the most distinct set of phyla as it is associated with 58 *Chloroflexi* BSVs and its well-known cyanobacterial symbiont *Cand.* Synechococcus spongiarum (BSV31). The network of archaeal *A. aerophoba* ASVs is small but shows similar phyla (*Crenarchaeota, Nanoarchaeota,* and *Thermoplasmatota*) to the other sponge.

### 3.6 Interaction between bacteria and archaea in the marine models

Archaeal association networks by themselves resulted in a random network with a maximum degree distribution of three and corresponding to the random Erdős-Réiny (ER) network. This is mainly due to the small number of ASVs after the prevalence cutoff (*n* = 32) (Supplementary Figures S7A,D left). The bacterial association network and the joint network show distinct clusters in comparison with the random ER network (Supplementary Figures S6B,D middle). Bacteria alone result in a network of 199 nodes with 588 edges, while the joint network is based on 231 nodes and 758 edges. Comparing the number of edges resulting from 100 iterations of the ER network, we find that these have a substantially higher number of edges (mean = 4,589, SD = 56) and degrees (mean = 39.83, SD = 5.77) when given the same clustering coefficient as the joint bacterial–archaeal network (Supplementary Figure S7E). This indicates that associations in the data driven network are based on true associations rather than random clustering. The bacterial community provides the main structure of the joint network as it provides the majority of BSVs (*n* = 199). Archaea (*n* = 32) are nested in close association with each other within the network. There are a total of six main modules in the network, which, except for one (red) are predominantly composed of bacterial associations (Figure 4C). The modules roughly correspond to the different hosts, namely, *M. leidyi* (purple), *H. panicea* (red), *A. aurita* (orange), and *A. aerophoba* (blue and yellow). Nodes that were identified as hubs or that have the highest centrality scores are of bacterial origin (Figure 4C; Supplementary Table S4) and belong to the *Chloroflexi* phylum associated with *A. aerophoba* (Figure 4C). Three of the five most connected nodes (highest degree) belong to the Archaea, namely, *Nitrosopumilus* and uncultured SCGC AAA286-E23. They all occur within the module resembling the community of *H. panicea* (Figure 4C middle; Supplementary Table S4), suggesting that taxa within this module interact closely with one another. Nodes with the highest betweenness represent keystone taxa between hosts. While they are present in both sponge hosts, their edges between the *A. aerophoba* and the *H. panicea* modules are predominantly negative correlations. This suggests that they may play different roles between modules. Most other modules are linked by positive correlations with each other, indicating that they are mutually exclusive, similarly to the indicator taxa (Figures 4A,B). The *H. panicea* symbiont *Amylibacter* has an average number of interaction partners (degree = 6) including *Nitrosopumilus* (ASV9), while *Cand.* Synechococcus spongiarum in *A. aerophoba* is more closely connected within its cluster (degree = 8) and has no connections to archaea.

## 4 Discussion

### 4.1 Insights and limitations for comparing microbiota in cross-models-system studies

Here, we present a multi-host study focusing on both the bacterial and the archaeal microbiome. As typically research focuses on only the bacterial community or individual hosts by themselves, studies covering multiple hosts and target bacteria and archaea are less common. Furthermore, by comparing both domains, we go one step further in identifying potential interaction partners within the microbiome. Challenges in studies, such as the one presented here, may arise due to differences in laboratory methods and the taxonomic databases. As described by Rausch et al. (2019), each host requires specialized protocols for DNA extraction, which have been trialed and tested within each working group to optimize extraction of the microbiota. Extractions are, therefore, not standardized across the different models. These may provide different results when breaking down cell membranes within and across different domains (Borrel et al., 2020). In addition, host evolutionary history may have an effect on the observed microbial community (Youngblut et al., 2019). Using Moran’s *I* (Gittleman and Kot, 1990), our data showed a weak positive phylogenetic autocorrelation only on the bacterial diversity (Shannon *H′*). This may be linked to the divergence between our terrestrial and aquatic hosts as within-group diversity was not significantly affected. It should be noted, however, that these data represent an approximation as divergence times were not available for all host species. While species divergence times do not seem to greatly affect our observations of the microbiota, the host environment may have a stronger impact on the community. This may particularly affect hosts, which interact strongly with their environment, including the aquatic hosts which are fully immersed in their environment and experience a strong exchange with the water column, or nematodes such as *C. elegans*, which recruit microbes from their substrates, such as rotting plant matter, known to harbor highly diverse communities (Johnke et al., 2020). While this was beyond the scope of this study, future in-depth analyses of the hosts should include samples of the immediate surroundings. This will allow for better distinction between persistent host–microbiota and taxa which may be brought in by ingestion or flow-through. Furthermore, the depth of taxonomic assignment may vary substantially between hosts, depending on the volume of research that has been put into isolation and characterization of the microbiota. It should also be noted that different taxonomies exist, which are continuously being updated (Parks et al., 2022). Here, we rely on the SILVA taxonomy, which represents a hybrid taxonomy between the most recent version of the GTDB and the NCBI databases. Discrepancies may therefore arise across taxonomic assignments in comparison with the more frequently updated GTDB. For ease of interpretations, we have therefore added differences in taxonomy in the main phyla to the figures. While host–archaea interactions identified here will need to be verified in the laboratory, they provide a starting point for research into the interactions of this domain with a variety of host models from different branches on the phylogenetic tree of life.

### 4.2 Archaea do not occur consistently across terrestrial hosts models

Six of the hosts studied here are terrestrial metaorganisms. The archaeome in terrestrial hosts has been of particular interest in a health-related context as they include humans and models for humans. We find that methanoarchaea such as *Methanobrevibacter smithii* and, to a lesser extent, *Methanomethylophilaceae* such as *Methanomassilicoccus* are most abundant in human stool (Figure 1A; Supplementary Figure S5). These results fall in line with previous descriptions of both the human archaeal and bacterial community (Lloyd-Price et al., 2019; Kurilshikov et al., 2021; Thingholm et al., 2021; Chibani et al., 2022). The archaeome in and on humans is generally well described (Koskinen et al., 2017; Pausan et al., 2019). They are present both in healthy adults, as in this study, but have also been associated with different disease phenotypes. Interestingly it has, however, not been possible to identify a single archaeon which follows a pathogenic lifestyle and has been shown to directly cause disease (Borrel et al., 2020; Runge and Rosshart, 2021). As host–microbiome interactions within the human metaorganism are notoriously difficult to study, microbiome research often uses simpler and/or more controllable host systems. Mice are commonly used as a proxy for human microbiota studies (reviewed in Runge and Rosshart, 2021), and mammals typically harbor methanoarchaea as an essential part of their gut microbial community (Youngblut et al., 2021). This, however, does not hold true for young mice where an archaeome seems to be absent. While humans and mice have a similar diversity and richness in their bacterial communities and share similar bacterial genera in their guts (Figures 1, 2), mice did not have an amplifiable archaeal community. This matches previous data analyses which were not able to detect this domain in mice (Beresford-Jones et al., 2022; Kieser et al., 2022). Studies using multi-host analysis to assess the archaeome also do not include data on mice, suggesting that no sufficient data were found (Youngblut et al., 2021; Thomas et al., 2022). Whether archaea remain below the detection limits of amplicons and metagenomes, are acquired at an older age, or are not present naturally is not known. However, a study comparing metagenomes in young rodents suggests that archaea are neither present in laboratory mice nor in wild populations (Bowerman et al., 2021).

Another, terrestrial host model is *C. elegans*. The archaeal community, which is less diverse than in humans (Figure 2), has thus far not been studied in this metaorganism. Our data show that *C. elegans* harbor *Methanobacterium* and *Methanosphaera*, similarly to humans (Figure 1A). We find that the archaeal community composition differs noticeably between slug and compost-derived *C. elegans*. This also applies to the bacterial community (Supplementary Figure S5), which can vary substantially based on their natural habitat in compost or slugs (Dirksen et al., 2016; Johnke et al., 2020; Pees et al., 2021). Easier manipulation of the host and its environment allows for analysis of microbiome-mediated evolution in mesocosms (Petersen et al., 2023), adaptation of microbes to the host (Obeng et al., 2023), or mechanisms of microbiome-mediated immune protection (Kissoyan et al., 2022). Considering that many archaea are anaerobes, this finding suggests that *C. elegans* provides a host environment which is suitable for anaerobic organisms and their potential function, as well as for aerobic organisms, as previously reported (Dirksen et al., 2016; Johnke et al., 2020). Moreover, our findings also identify *C. elegans* as future model system to study archaea–host interactions in a controlled setting.

While *C. elegans* looks like a promising candidate to study archaea, this cannot be said for the two arthropod models *D. melanogaster* and *G. mellonella*, which lack a replicable archaeal community. None of our samples passed our detection and filtering thresholds, suggesting that an archaeome is absent or present at only a very low abundance The bacterial diversity and richness are among the lowest found across our host models, corresponding to previous findings (Rausch et al., 2019). In the case of arthropods, it has been found that archaea and specifically methanoarchaea are often limited to detritivores (Tokura et al., 2000; Horváthová et al., 2021), indicating that the archaeome is directly linked to the insect food source. As a consistent core microbiome in *D. melanogaster* is thought to be absent (Wong et al., 2013), it has been recommended to limit microbiome analyses on flies to observations on microbiome dynamics in response to different interventions, rather than focusing on a description of the microbiota (Fink et al., 2013).

The last terrestrial model in this study is the vascular plant model *T. aestivum*. We find that its roots harbor the highest archaeal diversity across all tested hosts, an observation which may be attributed to its close association with soil. Plants often rely on highly complex microbial interactions within and around their roots and root nodules for the supply of nutrients from soil (Ofek et al., 2012). While beneficial effects of bacteria on growth and stress resistance are frequently tested, archaea are often overlooked from these analyses (Prudence et al., 2019). It therefore remains to be established whether they are similarly attracted by root exudates as beneficial bacteria are.

### 4.3 Microbial communities among marine hosts

The marine hosts covered by our study include one ctenophore, a cnidarian, and two porifera with evolutionarily ancient host–microbiota interactions. They are popular targets for microbe–host interaction studies as their relatively simple body structure facilitates research into these interactions (Weiland-Bräuer et al., 2015). Aquatic hosts often receive less attention than medically relevant model hosts, such as *M. musculus*, resulting in a bacterial and archaeal community that is less well covered by taxonomic databases. Our data reflect this in so far that many archaeal ASVs remain unclassifiable at the genus level and often above (Figure 1; Supplementary Figure S4). Comparing *M. leidyi* and *A. aurita,* we find that both archaeal and bacterial communities are distinct from one another with only three shared indicators, a fact which may be linked to their evolutionary history, as ctenophores are a phylum sister to all other animals, whereas sponges and cnidarians branch off at a later time (Schultz et al., 2023). Our results on the bacterial community composition of *M. leidyi* and *A. aurita* resemble previous comparative work on the two phyla (Weiland-Bräuer et al., 2015; Jaspers et al., 2020). Depending on their life stage, they, in this case *A. aurita*, also show a significant intra-host variability in their bacterial and also archaeal community composition (Figure 3; Supplementary Table S3; Supplementary Figure S4). While polyps are colonized by members of the family SCGC AAA286-E23, medusae harbor mainly *Nitrosopumilus,* which also feature as archaeal indicators (Figure 4). In the bacterial community, we find a high proportion of the *Marivita* genus, which is closely associated with the medusae, while polyps harbor an abundant unclassified *Gammaproteobacterium*. This matches previous observations on *A. aurita* developmental stages (Weiland-Bräuer et al., 2015), which have shown that development is impaired if the polyps lack their natural microbiota at the right time (Jensen et al., 2023). An interesting next step would be to investigate whether the archaeal component of their microbiota also plays an essential role in polyp development.

Porifera hosts include a high microbial abundance (HMA) sponge from the Mediterranean, *A. aerophoba,* and a low-microbial abundance (LMA) sponge from the Kiel bight, *H. panicea*. In agreement with HMA-LMA dichotomy in the bacterial community (Hentschel et al., 2006; Moitinho-Silva et al., 2017), we find bacterial diversity to be lower in the LMA sponge (Figure 2B). It is dominated by *Amylibacter* (Figure 4A), whereas the HMA sponge shows a more complex community composition (Figures 1B, 4A). *Amylibacters’* closest relative in the NCBI database is *Cand.* Halichondribacter symbioticus which has been shown to populate the mesohyl of *H. panicea* (Knobloch et al., 2020). We find that this symbiont has a medium number of interactions with other taxa (Figure 4), concurring with results by Schmittmann et al. (2022). Among its six interaction partners, we find one persistent colonizer of the HOC36 order (BSV70) as well as ASV9 assigned to the ammonia oxidizing *Nitrosopumilus* genus (Figures 4A,B). Together with *Cand.* Halichondribacter, they are involved in the removal and conversion of sponge waste products, such as ammonia (Knobloch et al., 2020). The low number of interactions with bacteria suggests that they are more reliant on exchange with the host than on the remaining microbiota. In opposition to LMA sponges, symbionts in HMA sponges are more diverse and their abundances are more evenly distributed. Commonly, they include the phylum *Chloroflexi* (Bayer et al., 2018), *Cand.* Synechococcus spongiarum, or *Cand.* Poribacteria (Slaby et al., 2017). The association networks show dense clustering around *Chloroflexi* hub nodes, as well as the *Cand.* S. spongiarum. This suggests that they interact more strongly with each other, for instance during the cycling of dissolved organic matter from *Cand.* S. spongiarum to *Chloroflexi* (Usher, 2008; Bayer et al., 2018).

Remarkably, we find that archaeal diversity is lower in the HMA sponge than in the LMA sponge (Figure 2), which is also reflected in the number of indicator taxa found per sponge species (Figure 4B). Archaeal diversity and richness, thus, show exactly the inverse trend to the common HMA-LMA dichotomy known for associated bacteria (Gloeckner et al., 2014; Moitinho-Silva et al., 2017). This could stem from different control mechanisms for bacteria and archaea in HMA and LMA sponges. If *A. aerophoba* has more control over its archaeal community composition, it may harbor archaeal members more specific for this host, as is suggested by the negative associations between *Aplysina-*associated ASVs and the larger clusters linked to the other three marine hosts (Figure 4). However, similarly to bacterial communities, both HMA and LMA sponges are known to harbor sponge-specific archaea that differ from the surrounding seawater (Chaib De Mares et al., 2017; Steinert et al., 2020; Polónia et al., 2021) and that are adapted to the sponge-host lifestyle (Haber et al., 2020; Wang et al., 2022). Therefore, both *A. aerophoba* and *H. panicea* are expected to actively control archaea and to be dependent on these symbioses. With a less diverse bacterial microbiome, *H. panicea* might be more dependent on these functions and metabolites provided by the associated archaeal community and thus select for a more diverse archaeal community. It should be noted, however, that a study comparing *A. aerophoba* to other LMA sponges found similar archaeal diversity between species (Chaib De Mares et al., 2017). Furthermore, the relative abundances and diversity we discussed here may differ from absolute abundances. Archaeal abundances quantified by qPCR for several sponge species including *A. aerophoba* correspond to the HMA-LMA dichotomy (Bayer et al., 2014). At this point, we cannot distinguish whether the patterns we observe here are specific to the sponge species or related to their bacterial HMA-LMA status. Future studies could therefore benefit from inclusion of the surrounding seawater, as well as the assessment of microbial ratios through qPCR or metagenome sequencing.

### 4.4 Archaea in marine metazoan nutrient cycling

The marine hosts analyzed in this study were found to harbor predominantly archaea from the *Nitrosopumilus* genus, which are ubiquitous in the marine environment and have previously been observed in association with sponges (Bayer et al., 2008; Chaib De Mares et al., 2017; Steinert et al., 2020). *Nitrosopumilus* have been shown to be essential players as ammonia oxidizers in the marine nitrogen cycle (Francis et al., 2007; Wright and Lehtovirta-Morley, 2023). We find that they are associated with other nitrogen cyclers, such as *Marivita* which can reduce oxidized nitrogen compounds during denitrification (Zheng et al., 2019) and are affiliated with *A. aurita*. A further interaction partner is *Nitrosomonas* (BSV1013) which are thought to contribute to nitrification in the sponge mesohyl (Hentschel et al., 2006; Bayer et al., 2008). Potential ammonia sources, such as cyanobacteria, including the *A. aerophoba* symbiont *Cand.* Synechococcus spongiarum (BSV31), do not associate with archaea in our analysis (Figure 4C). This role may fall to the sponge as it can provide ammonia by itself (Bayer et al., 2008). *Nitrosopumilus* have also been found to be able to produce oxygen from nitrate under oxygen limited conditions (Kraft et al., 2022). This may provide an additional advantage for survival in the sponge mesohyl, which undergoes diurnal fluctuations in oxygen as reviewed by Zhang et al. (2019).

Other abundant archaea associated with ctenophores, cnidaria, and sponges belong to the *Woesearchaeales* and individual *Thermoplasmata* affiliated with the prevalent Marine Group II (Figure 4). Little is currently known about the uncultured order of the *Woesearchaeales*. Recent research on metagenome assembled genomes shows that they predominantly occur in anoxic environments where they may provide hydrogen to methanoarchaea in a syntrophic relationship (Liu et al., 2018). *Woesearchaeota* and the SCGC AAA286-E23 have also been detected in the seagrass phyllosphere (Vogel et al., 2021) but are only rarely detected in the marine water column. While they may be adapted to anaerobic niches within the marine hosts, they associate mostly with *Nitrosopumilus* and aerobic *Gammaproteobacteria* in our dataset. Marine Group II archaea may supply nutrients to our marine hosts as they are thought to be involved in the initial breakdown of organic matter, peaking after algal blooms (Zhang et al., 2015; Jain and Krishnan, 2021). However, no member of this group has been isolated to date. As taxonomic resolution of *Woesearchaeales* and Marine Group II remains inexact and undergoes regular reconstruction (Parks et al., 2022), and isolates are still amiss; further research will be needed to discover their taxonomy and role in the metaorganism.

### 4.5 Diversity and richness between terrestrial and aquatic hosts

Most hosts in this study show a substantially higher bacterial diversity and richness than in the archaeal community. Grouping the hosts by either terrestrial or aquatic environment, we find that only bacterial richness is slightly significantly higher in the aquatic hosts. This trend in the bacterial community is far less pronounced here in comparison with the cross-model study by Rausch et al. (2019), where terrestrialization was hypothesized to represent a key evolutionary event for the diversification of host-associated microbiota. Differences in our study may arise particularly due to *H. panicea*, whose high archaeal diversity is responsible for a change in overall group means and differs from our initial expectations. In cases such as this, the low bacterial diversity is linked to the high abundance of a single taxon, which persists in the host and is often passed on directly to its offspring (Schmittmann et al., 2022; Carrier et al., 2023). Variability in symbiont presence/absence and abundance can occur among different host populations but also different sampled body parts. For instance, *D. melanogaster* communities often show *Wolbachia,* which are transmitted through their germline (Simhadri et al., 2017). In this case, we used a Wolbachia-free strain explaining the absence of the symbiont; however, the use of extraction protocols in which only the intestine or even only the feces are analyzed can also substantially reduce *Wolbachia* signals as the majority of *Wolbachia* endosymbionts are found in the organs of the germline (Fink et al., 2013). Invertebrates, including ctenophores and cnidarians, show species and even population specific differences regarding the presence or absence of highly abundant bacterial taxa (Fraune and Bosch, 2007; Weiland-Bräuer et al., 2015; Jaspers et al., 2020). While we find recurring taxonomic groups in the marine hosts, particularly among the archaea, their ASVs tend to cluster separately from each other. In the case of *Nitrosopumilus* associated with *A. aerophoba* or the other marine hosts, association is even negative, suggesting that these may be sponge-specific variants which have co-evolved with their hosts (Theis et al., 2016; Chaib De Mares et al., 2017).

An aspect that may significantly influence host diversity, regardless of its aquatic or terrestrial origin, may be the level of domestication. Hosts addressed in this study generally show very different levels of domestication. They range from freshly caught *M. leidyi* and sponges to wild-derived but lab-maintained mouse subspecies, and long-standing laboratory populations of *H. vulgaris*. Hosts, which have a long history of domestication or rearing in the laboratory, such as lab-reared mice, often show a decrease in their bacterial diversity (Rosshart et al., 2019; Bowerman et al., 2021). The same has been observed in plants which often experience a loss of bacterial diversity in association with domestication (Hassani et al., 2020; Özkurt et al., 2020). Here, this trend may also apply to the microbial community of *H. vulgaris* as it represents a long-standing laboratory model and completely lacks a detectable archaeal community. Further research is, however, needed to understand whether archaea are similarly affected by domestication than host-associated bacteria.

## 5 Conclusion

Taken together, we find that archaeal diversity is significantly lower than the corresponding bacterial diversity across most studied hosts. Contrary to our expectation, archaeal diversity and richness did not differ significantly between aquatic and terrestrial hosts. The bacterial communities tended to be richer in the aquatic models, confirming previous findings. While the genus *Nitrosopumilus* is highly prevalent among the archaeal community, their ASVs are taxon-specific, indicating specific adaptations to their hosts. Archaeal ASVs show a particularly high betweenness but negative association between host clusters, suggesting that they may take on similar key roles within their host, which are functionally redundant between them. Their role in marine hosts may be linked to nitrogen cycling as the abundant *Nitrosopumilus* genus has previously been identified to be an ammonia oxidizer. Further interpretation of archaeal functions, however, is limited due to coarse taxonomic assignment and the lack of cultured and characterized isolates. This applies especially to the *Nanoarchaeota* as only a low number of cultured isolates and information currently exist in the public databases. Further efforts in the isolation and characterization of archaea are urgently needed to improve our understanding of their role in the metaorganism.

## Data availability statement

Original datasets are deposited in the NCBI SRA under BioProject accession number PRJNA1045552: https://www.ncbi.nlm.nih.gov/sra.

## Ethics statement

The studies involving humans were approved by the Ethics committee at Kiel University (AZ A103/14). The studies were conducted in accordance with the local legislation and institutional requirements. The participants provided their written informed consent to participate in this study. The animal study was approved by the Veterinäramt Kreis Plön permit: 1401–144/PLÖ–004697. The study was conducted in accordance with the local legislation and institutional requirements.

## Author contributions

CB: Conceptualization, Methodology, Resources, Writing – review & editing. PR: Conceptualization, Methodology, Validation, Writing – review & editing. MR: Investigation, Resources, Writing – review & editing. HF: Investigation, Writing – review & editing. JH: Investigation, Writing – review & editing. NJ: Investigation, Writing – review & editing. MK: Investigation, Writing – review & editing. CP: Investigation, Writing – review & editing. LS: Investigation, Writing – review & editing. TZ: Investigation, Writing – review & editing. JB: Writing – review & editing. TB: Writing – review & editing. UH: Writing – review & editing. TBHR: Writing – review & editing. TR: Writing – review & editing. AF: Writing – review & editing. HS: Writing – review & editing. ES: Conceptualization, Project administration, Writing – review & editing. RS: Conceptualization, Funding acquisition, Project administration, Writing – review & editing. AH-H: Conceptualization, Data curation, Formal Analysis, Investigation, Visualization, Writing – Original draft.

## Footnotes

^1^https://www.metaorganism-research.com/

## Supplementary material

SUPPLEMENTARY TABLE S1 Read counts and amplicon sequence variants during processing and quality control of the archaeal and bacterial datasets.

SUPPLEMENTARY TABLE S2 Results of the pairwise PERMANOVA analysis on Bray-Curtis distances between hosts.

SUPPLEMENTARY TABLE S3 Results of the pairwise PERMANOVA analysis on Bray-Curtis distances within marine host sub-samples.

SUPPLEMENTARY TABLE S4 Network properties from the network of archaeal and bacterial ASVs in NetCoMi, including clusters, hubs, degrees and centrality scores.
