# Supplementary material for: The archaeome in metaorganism research, with a focus on marine models and their bacteria–archaea interactions
Source: Front Microbiol. 2024 Feb 27;15:1347422. doi: 10.3389/fmicb.2024.1347422 (PMC10927989; doi:10.3389/fmicb.2024.1347422)
Supplement: Supplementary file 5 [file Data_Sheet_1.docx]

Supplementary Material


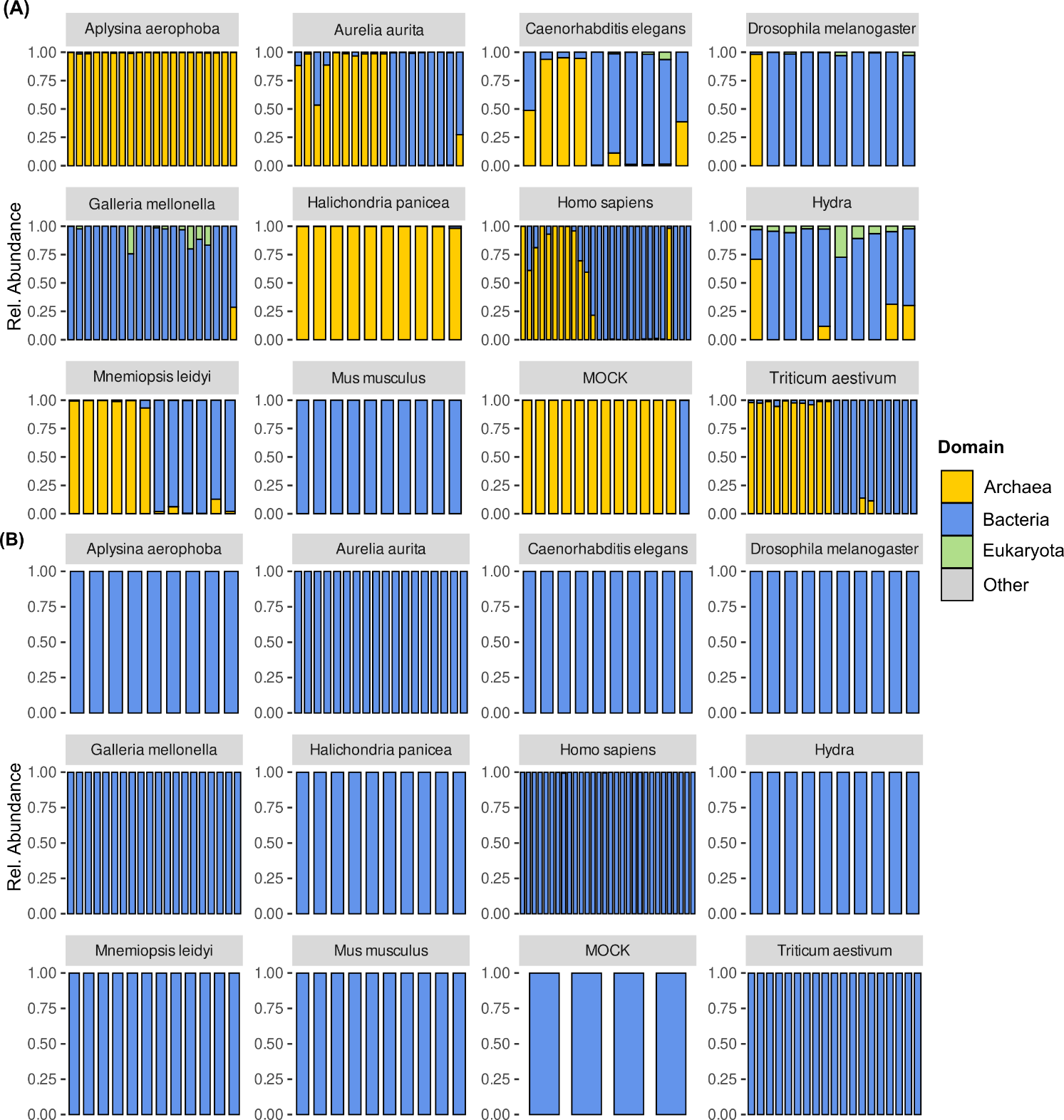


Supplementary Figure S1: Distribution of kingdoms and extrinsic domains (other) in the raw amplicon data. The barcharts are based on relative abundances of the raw reads in the archaeal (A) and bacterial (B) dataset plotted as proportion of 1, where 1=100%. Each bar represents one sample within each host. The category “Other “ includes mitochondria and chloroplasts.


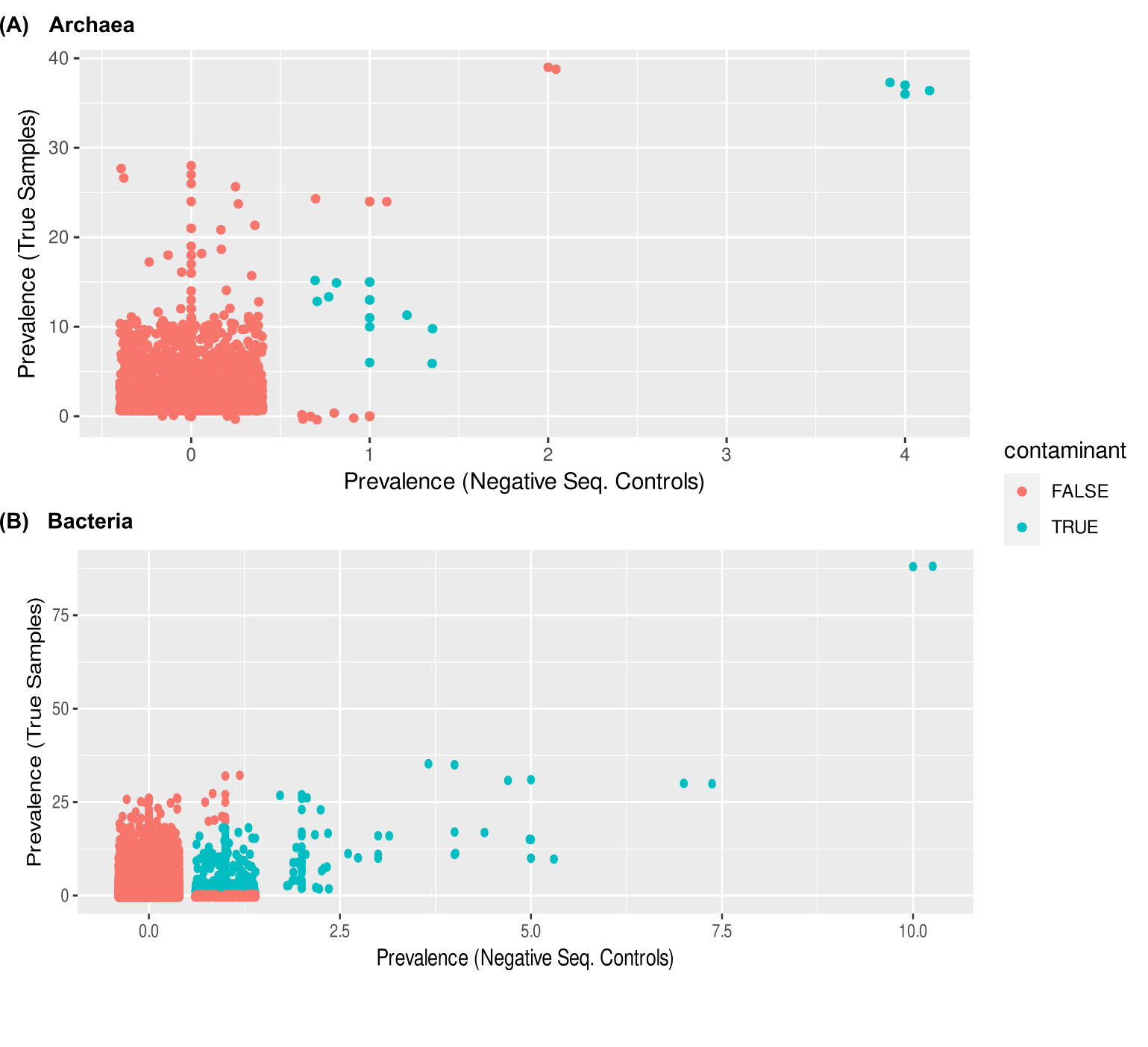


Supplementary Figure S2: Archaeal (A) and bacterial (B) contaminants identified by decontam. Contaminants were identified using statistical decontamination against all available negative controls (sequencing and extraction blanks) with a prevalence threshold of 0.5.


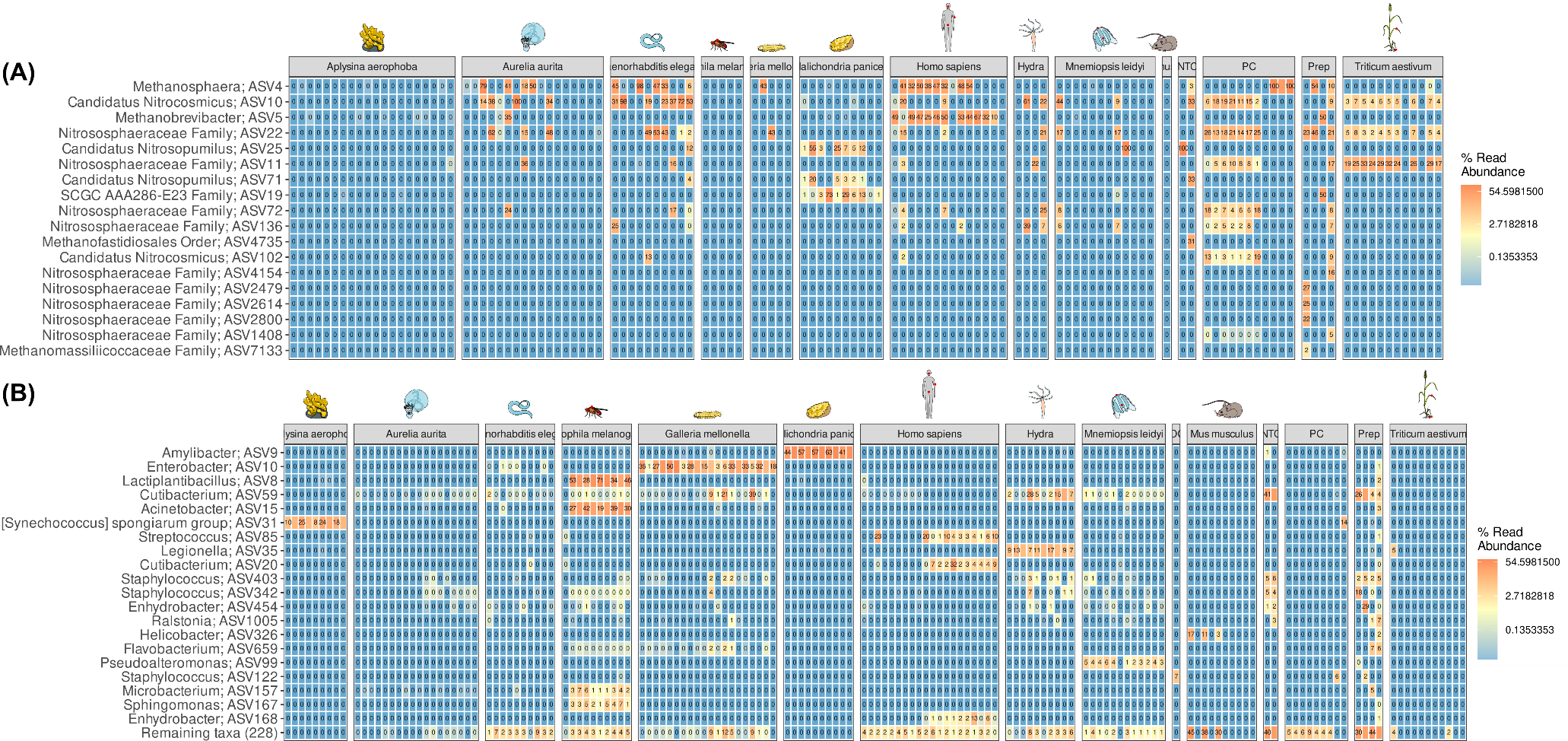


Supplementary Figure S3: Most abundant archaeal (A) and bacterial (B) contaminants. Contaminants were identified using statistical decontamination with the decontam package (Davis et al. 2018) against all available negative controls (sequencing (NTC) and extraction blanks (Prep)) with a prevalence threshold of 0.5.


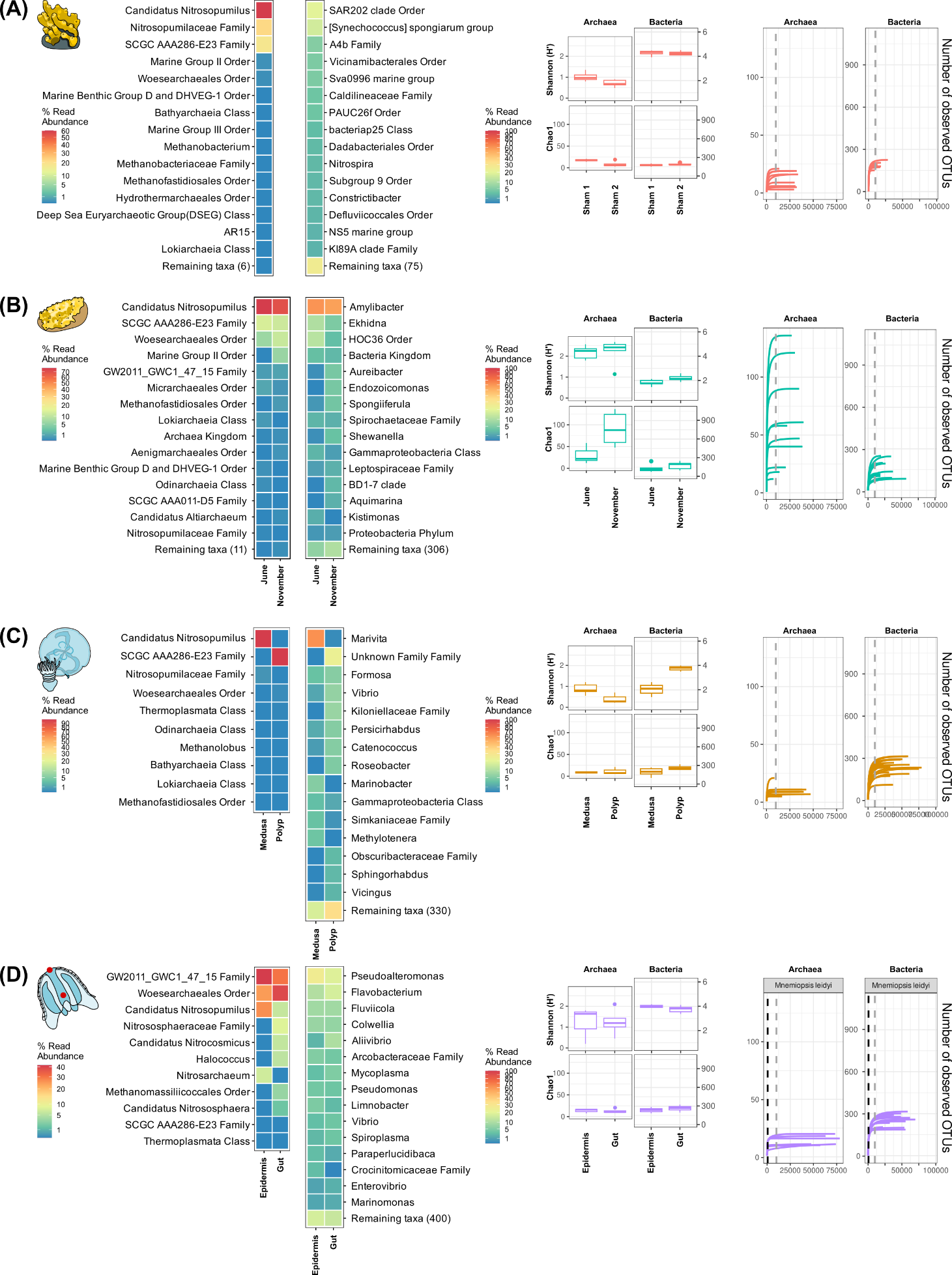


Supplementary Figure S4 Heatmaps reflecting the most abundant archaeal and 15 most abundant bacterial taxa, diversity and richness for each sample type, as well as rarefaction curves for marine hosts *A. aerophoba* (A), *H. panicea* (B), *A. aurita* (C) and *M.leidyi* (D). All samples after quality filtering and with more than 500 raw reads were included. Diversity and richness indices were calculated based on reads rarefied at 10.000. Dashed vertical lines in the rarefaction plots indicate the rarefaction cutoff of 10.000.


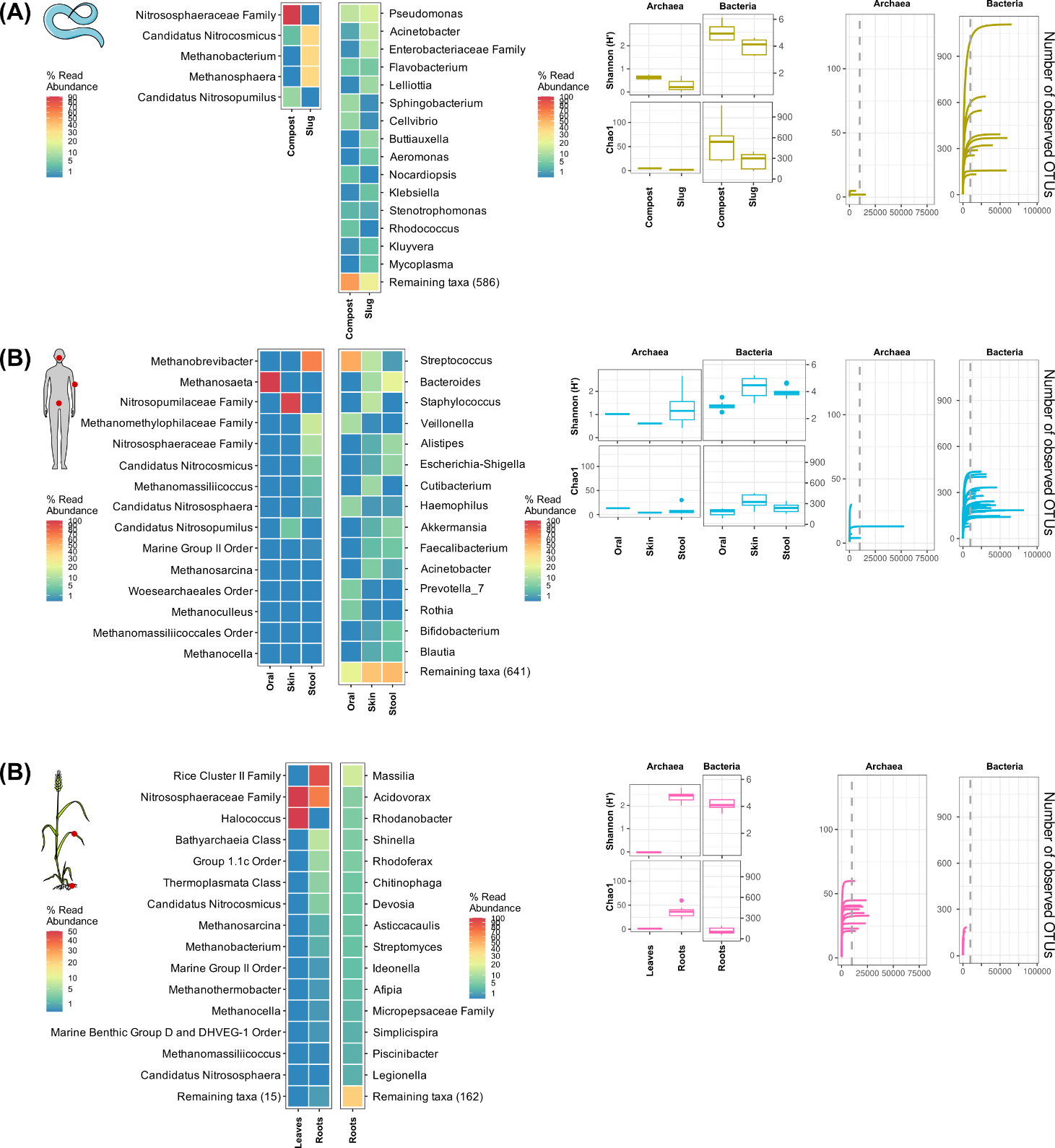


Supplementary Figure S5 Heatmaps reflecting the most abundant archaeal and 15 most abundant bacterial taxa, diversity and richness for each sample type, as well as rarefaction curves for hosts *C. elegans* (A), *H. sapiens* (B) and *T. aestivum* (C). All samples after quality filtering and with more than 500 raw reads were included. Diversity and richness indices were calculated based on reads rarefied at 10.000. Dashed vertical lines in the rarefaction plots indicate the rarefaction cutoff of 10.000.


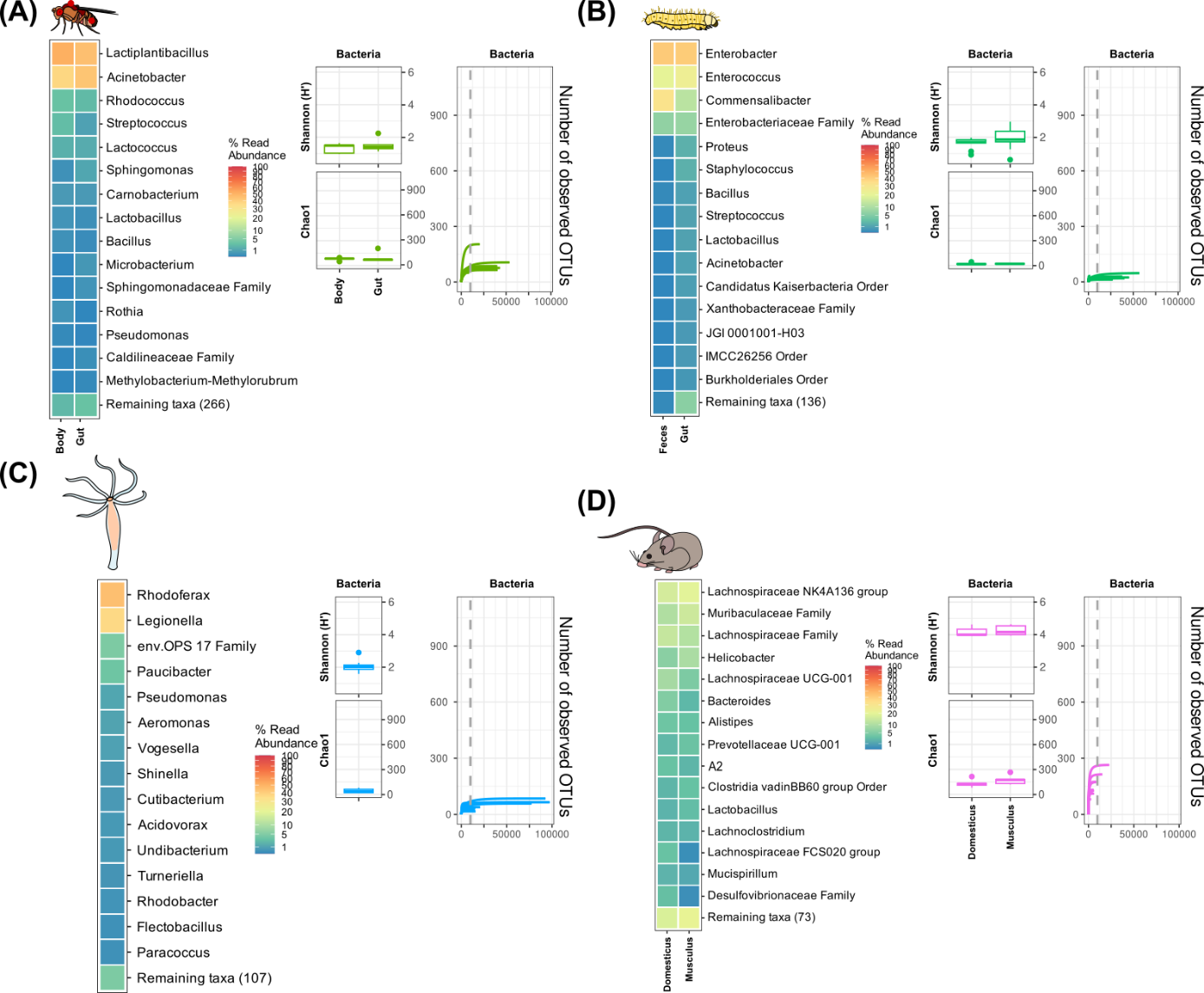


Supplementary Figure S6 Heatmaps reflecting the most abundant archaeal and 15 most abundant bacterial taxa, diversity and richness for each sample type, as well as rarefaction curves for hosts *D. melanogaster* (A), *G. melonella* (B) and *H. vulgaris* (C) and *M. musculus* (D). All samples after quality filtering and with more than 500 raw reads were included. Diversity and richness indices were calculated based on reads rarefied at 10.000. Dashed vertical lines in the rarefaction plots indicate the rarefaction cutoff of 10.000.


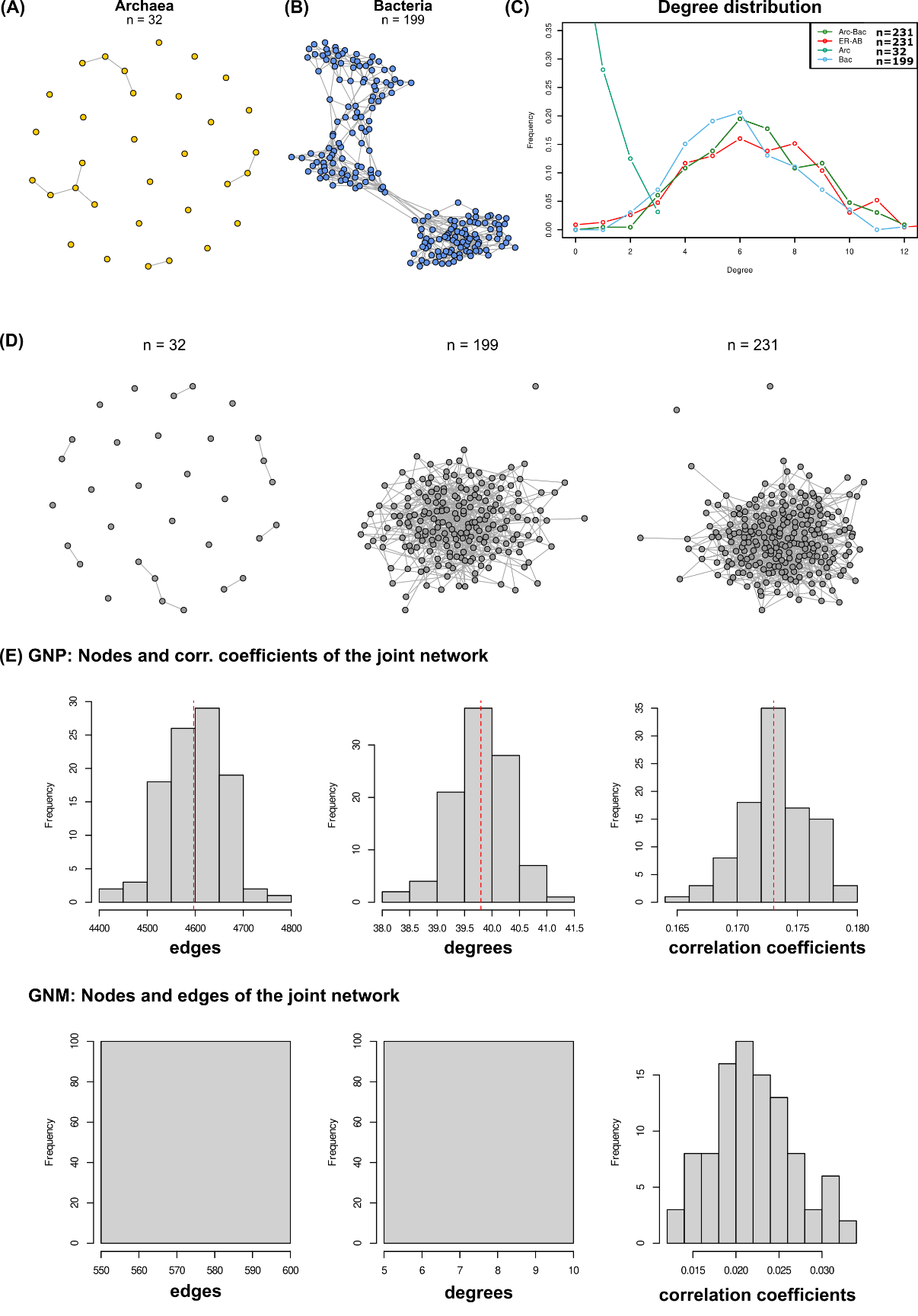


Supplementary Figure S7 Base networks for archaea and bacteria, degree distributions and Erdős-Rényi (ER) graphs and statistics. Individual networks were calculated for archaea (A) and bacteria (B) using SpiecEasi. C) The degree distributions reflect the ER network corresponding to the joint bacterial-archaeal network, as well as the individual networks. E) Random ER-networks corresponding to Archaea, Bacteria, and the joint network. Networks were calculated using the same number of edges and nodes. E) Network statistics on 100 random networks using Erdős-Rényi graphs and either the same node number and correlation coefficient of the joint bacteria-archaea network (GNP) or the number of nodes and edges from this network (GNM). The red dashed lines indicate mean values across the 100 replications.
